# MUC1-C integrates aerobic glycolysis with suppression of oxidative phosphorylation in triple-negative breast cancer stem cells

**DOI:** 10.1016/j.isci.2023.108168

**Published:** 2023-10-11

**Authors:** Nami Yamashita, Henry Withers, Yoshihiro Morimoto, Atrayee Bhattacharya, Naoki Haratake, Tatsuaki Daimon, Atsushi Fushimi, Ayako Nakashoji, Aaron R. Thorner, Emily Isenhart, Spencer Rosario, Mark D. Long, Donald Kufe

**Affiliations:** 1 Dana-Farber Cancer Institute, Harvard Medical School, Boston, MA, USA; 2 Department of Biostatistics & Bioinformatics, Roswell Park Comprehensive Cancer Center, Buffalo, NY, USA

**Keywords:** Molecular biology, Cell biology, Omics, Transcriptomics

## Abstract

Activation of the MUC1-C protein promotes lineage plasticity, epigenetic reprogramming, and the cancer stem cell (CSC) state. The present studies performed on enriched populations of triple-negative breast cancer (TNBC) CSCs demonstrate that MUC1-C is essential for integrating activation of glycolytic pathway genes with self-renewal and tumorigenicity. MUC1-C further integrates the glycolytic pathway with suppression of mitochondrial DNA (mtDNA) genes encoding components of mitochondrial Complexes I–V. The repression of mtDNA genes is explained by MUC1-C-mediated (i) downregulation of the mitochondrial transcription factor A (TFAM) required for mtDNA transcription and (ii) induction of the mitochondrial transcription termination factor 3 (mTERF3). In support of pathogenesis that suppresses mitochondrial ROS production, targeting MUC1-C increases (i) mtDNA gene transcription, (ii) superoxide levels, and (iii) loss of self-renewal capacity. These findings and scRNA-seq analysis of CSC subpopulations indicate that MUC1-C regulates self-renewal and redox balance by integrating activation of glycolysis with suppression of oxidative phosphorylation.

## Highlights

• Enriched CSCs are dependent on MUC1-C for self-renewal capacity and tumorigenicity
• MUC1-C upregulates genes encoding GLUT1, HK2, PGAM1, and other glycolytic enzymes
• MUC1-C represses mitochondrial DNA genes encoding Complexes I–V of the ETC
• CSCs maintain redox balance by MUC1-C-driven regulation of aerobic glycolysis and OXPHOS

## Graphical abstract

**Figure:**
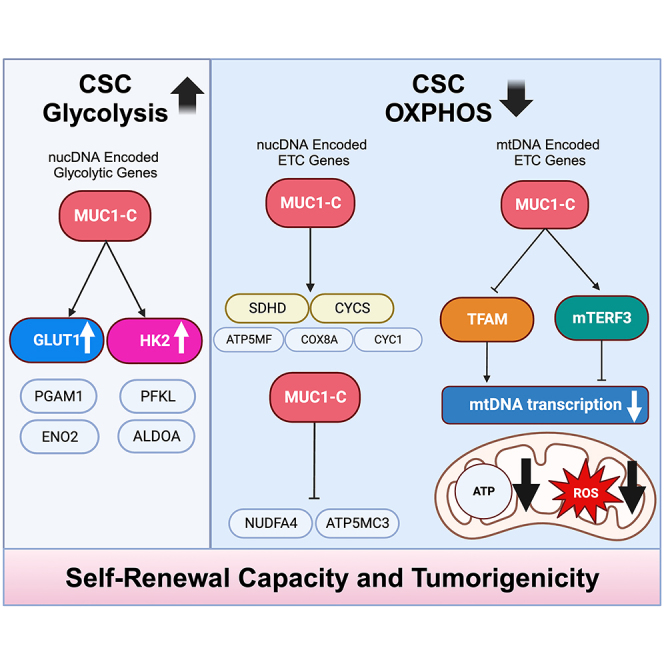

## Introduction

The *MUC1* gene evolved in mammals to protect barrier tissues from the external environment.^1^^,^^2^ *MUC1* encodes two subunits that form a heterodimer at the apical cell membrane.^1^^,^^2^ The MUC1 N-terminal (MUC1-N) subunit extends beyond the glycocalyx into a mucous gel that functions as a physical barrier.^1^^,^^2^ The transmembrane MUC1 C-terminal (MUC1-C) subunit is activated by loss of homeostasis and induces inflammatory, remodeling, and repair responses associated with wound healing.^1^^,^^2^ As a result, MUC1-C is imported from the cell membrane to the nucleus, where it interacts with proinflammatory transcription factors (TFs), such as NF-κB and STAT3, and contributes to the regulation of their target genes.^3^^,^^4^ MUC1-C thereby induces the epithelial-mesenchymal transition (EMT) by driving expression of the ZEB1, TWIST1, and SNAIL EMT TFs.^5^ Nuclear MUC1-C also interacts with NF-κB, MYC, and E2F in driving PRC1/2 complexes and epigenetic reprogramming.^6^ In addition, nuclear MUC1-C interacts with E2F1 in activating the SWI/SNF BAF and PBAF chromatin remodeling complexes^7^^,^^8^ and global changes in chromatin architecture.^9^^,^^10^ In principle, these changes are reversible with reestablishment of homeostasis; however, prolonged activation of MUC1-C in settings of chronic inflammation promotes pan-cancer progression.^1^^,^^2^^,^^10^ Along these lines, in TNBC and other types of recalcitrant carcinomas, MUC1-C induces lineage plasticity, self-renewal capacity, and tumorigenicity in concert with driving the cancer stem cell (CSC) state.^5^^,^^11^^,^^12^^,^^13^^,^^14^^,^^15^^,^^16^ Consistent with these findings, MUC1 expression associates poor clinical outcomes across pan-cancers.^17^

TNBCs are enriched in CSCs that contribute to DNA damage resistance, immune evasion, and poor clinical outcomes.^16^^,^^18^^,^^19^ TNBC progression is driven at least in part by metabolic reprogramming^20^^,^^21^; however, little is known in regard to the regulation of TNBC CSC metabolism. Certain TNBC tumors exhibit elevated glucose uptake and a glycolytic gene signature.^22^ In addition, TNBC cell lines grown as monolayers in two-dimensional (2D) culture are dependent on high levels of glycolysis.^23^ As substantiated by this metabolic profile, TNBC tumors and cell lines exhibit upregulation of the GLUT1 glucose transporter, whereas expression of other effectors in the glycolytic pathway is largely heterogeneous.^24^ Downstream to GLUT1, hexokinase 2 (HK2) functions as the rate-limiting enzyme that catalyzes the first step of glycolysis.^25^ Less is known about HK2 expression in TNBC cells, although HK2 is often upregulated in other human cancers and drives their reprogramming to aerobic glycolysis.^25^ Cancer cells are not exclusively dependent on aerobic glycolysis in that mitochondria also play important roles in growth and survival.^26^^,^^27^ Indeed, TNBC cells are at least in part dependent on mitochondrial function.^20^^,^^28^ Oxidative phosphorylation (OXPHOS) is upregulated in TNBC cells with RB deficiency or induction of E2F1,^29^ indicating that oncogenic signaling pathways may dictate reliance on glycolytic vs. OXPHOS activities.

MUC1-C is a pan-cancer oncogenic protein^1^^,^^2^^,^^10^ that has been linked to certain pathways of metabolic regulation.^30^^,^^31^^,^^32^^,^^33^ As one example, MUC1-C activates TP53-inducible regulator of glycolysis and apoptosis (TIGAR) and the pentose phosphate pathway (PPP).^34^ These studies were performed on TNBC and other types of cancer cells grown in 2D culture and thus may not reflect involvement associated with the CSC state. In this way, enriched TNBC CSCs selected by serial passage of mammospheres represent a potential model for identifying MUC1-C dependencies that integrate metabolic reprogramming with self-renewal capacity. The present studies demonstrate that MUC1-C is necessary for activation of the aerobic glycolytic pathway and suppression of OXPHOS in driving the TNBC CSC state.

## Results

### Serially passaged TNBC CSCs are dependent on MUC1-C for self-renewal

**Figure 1:**
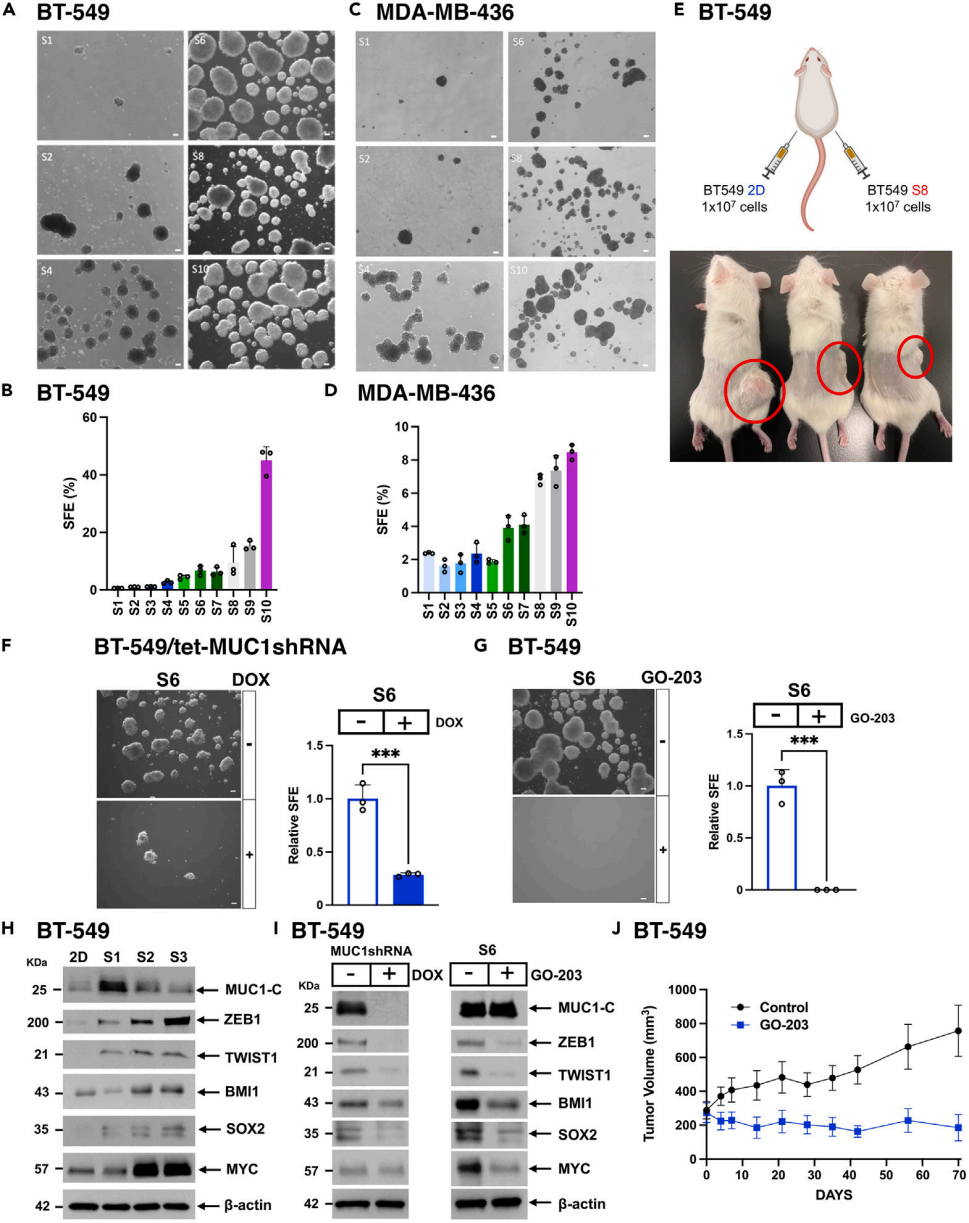
Serial passage of TNBC mammospheres selects for MUC1-C-dependent CSC populations (A and B) BT-549 cells growing as monolayers were seeded in mammosphere culture medium. After 10 days, the sphere 1 (S1) cells were isolated and reseeded for selection of S2 cells. Photomicrographs are shown for the serially passaged mammospheres up to S10. Scale bar: 100 μm. (A). The sphere forming efficiency (SFE) was determined by the percentage of cells that formed mammospheres as a function of the number of seeded cells. SFE is expressed as the % (mean ± SD of three determinations) (B). (C and D) MDA-MB-436 cells serially passaged as mammospheres are shown in the indicated S1-S10 photomicrographs. Scale bar: 100 μm. (C). SFE is expressed as the % (mean ± SD of three determinations) (D). (E) BT-549 2D and S8 mammosphere cells (10 × 10^6^) were implanted into left and right flanks, respectively, of NSG mice. Shown are tumors at 5 months after implantation. (F) BT-549/tet-MUC1shRNA 3D cells treated with vehicle or DOX for 7 days were analyzed for sphere formation. Shown are photomicrographs of representative mammospheres. Scale bar: 100 μm. (left). The relative SFE is expressed as the mean ± SD of three determinations as compared to that obtained for vehicle-treated cells (assigned a value of 1) (right). Asterisks represent  ∗∗∗p ≤ 0.001. (G) BT-549 3D cells were treated with vehicle or 2.5 μM GO-203 for 3 days. Shown are photomicrographs of representative mammospheres. Scale bar: 100 μm (left). The relative SFE is expressed as the mean ± SD of three determinations as compared to that obtained for vehicle-treated cells (assigned a value of 1) (right). Asterisks represent ∗∗∗p ≤ 0.001. (H) Lysates from BT-549 cells grown in 2D culture and as serially passage mammospheres (S1-S3) were immunoblotted with antibodies against the indicated proteins. (I) Lysates from BT-549/tet-MUC1shRNA 3D cells treated with vehicle or DOX for 10 days were immunoblotted with antibodies against the indicated proteins (left). Lysates from BT-549 3D cells were treated with vehicle or 2.5 μM GO-203 for 3 days were immunoblotted with antibodies against the indicated proteins (right). (J) NSG mice with established BT-549 3D cell tumors were treated intraperitoneally with PBS or GO-203 (12 μg/gm body weight) each day for 70 days. Tumor volumes are expressed as the mean ± SEM for 6 tumors.

Studies of TNBC CSCs have been hampered by challenges using cell surface markers in purifying this cell population.^35^^,^^36^ Nonetheless, isolation of CSCs as defined functionally has been advanced by growth in serum-free conditions to maintain the undifferentiated CSC state.^37^^,^^38^ Accordingly, we enriched TNBC CSC populations based on the functional capacity to form mammospheres. Using this approach, BT-549 TNBC cells growing in 2D culture were established as serial passage 1 (S1) mammospheres (Figure 1A). These mammosphere cells (>100 μm) were then isolated and serially passaged for up to S10 mammospheres (Figure 1A). With each passage, we observed progressive increases in sphere size (Figure 1A), sphere forming efficiency (SFE) as the percentage of functional CSCs (Figure 1B), and proliferation rate (Figure S1A). As a second model, similar results were obtained with MDA-MB-436 TNBC cells; that is, increases in mammosphere size (Figure 1C), SFE (Figure 1D), and proliferation rate (Figure S1B). BT-549 cells are recognized for lacking the capacity to form tumors in immunocompromised mice. The basis for this deficiency has remained unclear. Nonetheless, implanting BT-549 S8 sphere cells in 3 of 3 NSG mice resulted in the formation of tumor xenografts (Figure 1E), indicating that enrichment of BT-549 cells for anchorage-independent growth increases their propensity for forming tumors, albeit not as efficiently as in other cancer models. In addition, serial passage of fragments from these founding xenografts readily formed tumors for further study (Figure S1C). Unlike BT-549 cells, MDA-MB-436 cells grown as 2D monolayers form tumors in nude mice. We therefore examined the effects of serial dilutions on the capacity of MDA-MB-436 2D and S8 cells to establish tumors. The results showed that MDA-MB-436 S8 sphere cells are more efficient in forming tumors (Figure S1D), further supporting the selection of TNBC CSCs as defined functionally in serially passaged mammospheres. MUC1-C promotes lineage plasticity and self-renewal of TNBC cells grown in 2D culture^5^; whereas, it is not known if serially passaged TNBC CSCs have similar dependencies. We found that silencing MUC1-C in BT-549 and MDA-MB-436 cells suppresses self-renewal capacity as evidenced by decreases in mammosphere formation (Figures 1F, S1E, and S1F). In confirming that MUC1-C is necessary for CSC self-renewal, we used a second MUC1shRNA#2 which similarly decreased mammosphere formation (Figures S1G and S1H). In addition, rescuing MUC1-C expression with a tet-MUC1-C cytoplasmic domain (MUC1-C/CD) expressing vector (Figure S1I) reestablished the capacity for forming mammospheres (Figure S1J). MUC1-C/CD includes a CQC motif that is required for MUC1-C dimerization, nuclear localization and oncogenic function.^1^ Treatment of BT-549 and MDA-MB-436 cells with the GO-203 inhibitor, which blocks the CQC motif, markedly suppressed mammosphere formation (Figures 1G and S1K), further indicating that MUC1-C is necessary for CSC self-renewal. Along these lines, MUC1-C expression was increased in lysates from BT-549 3D (S1) vs. 2D cells (Figure 1H). With serial passage (S3), MUC1-C levels declined to those observed in 2D cells (Figure 1H) coincident with increases in the chromatin fraction. Subsequent studies are therefore underway to assess the functional significance of MUC1-C localization to chromatin in association with progression of the CSC state. In regard to CSC markers, CD44^high^/CD24^low^ cell surface expression has been used to isolate BC CSCs^39^; however, more recent work has indicated this population is a heterogeneous spectrum of EMT phenotypes.^36^ Compared to 2D cells, serially passaged 3D CSCs exhibited upregulation of the (i) ZEB1 and TWIST1 EMT-TFs that contribute to the CSC state,^36^ (ii) BMI1/PRC1 stemness marker,^40^ and (iii) SOX2 and MYC, which have also been linked to driving TNBC CSCs^41^^,^^42^(Figure 1H). Significantly, expression of these effectors in CSCs was suppressed by silencing MUC1-C (Figure 1I, left) and by targeting MUC1-C with GO-203, which blocks MUC1-C function in the absence of having an effect on MUC1-C levels (Figure 1I, right). Moreover, GO-203 treatment of mice bearing passaged BT-549 CSC xenografts inhibited tumor growth (Figures 1J and S1L), confirming that MUC1-C is necessary for self-renewal and tumorigenicity.

### MUC1-C regulates glycolytic genes in TNBC CSCs

**Figure 2:**
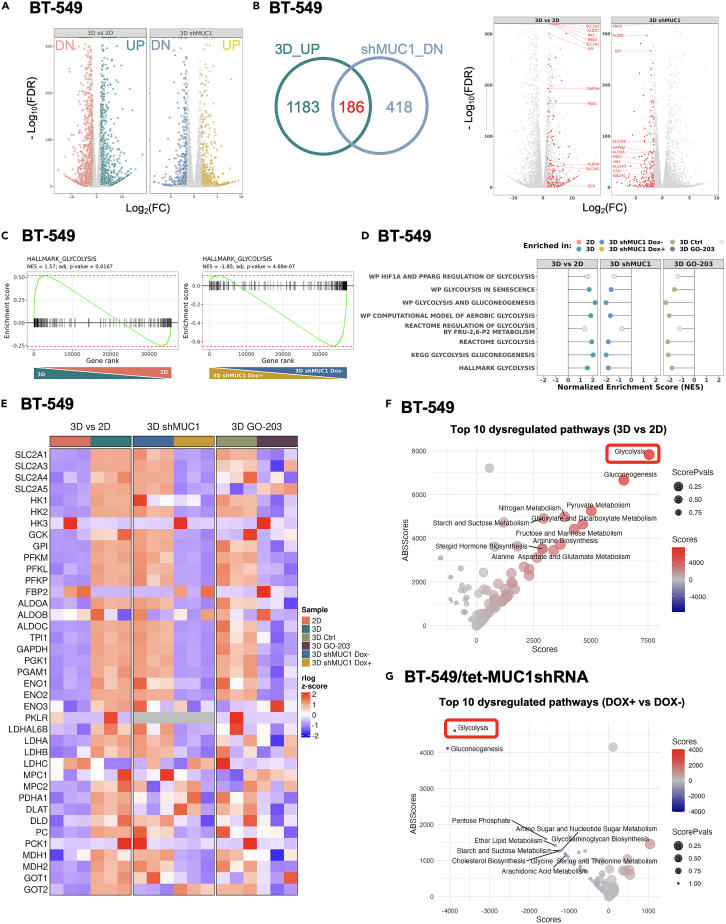
MUC1-C is necessary for activation of glycolysis gene signatures in CSCs (A) Volcano plots showing downregulated (left) and upregulated (right) genes in BT-549 cells grown in S6 3D vs. 2D culture (left; 3070 DEGs; <0.1 padj (Benjamini-Hochberg adjusted p value), log2fold change (FC) > 1.5), with 1,369 upregulated and 1,701 downregulated genes). Volcano plots of downregulated (left) and upregulated (right) genes in BT-549/tet-MUC1shRNA 3D cells treated with DOX vs. vehicle for 10 days (right; 1678 DEGs; <0.1 padj (Benjamini-Hochberg adjusted p value), log2fold change ((FC) > 1.5), with 1,074 upregulated and 604 downregulated genes). (B) Overlap of DEGs upregulated in 3D BT-549 cells and downregulated in 3D BT-549/tet-MUC1 shRNA cells treated with DOX (left). The differential expression of these shared genes (red) is shown for the two conditions (right). Glycolytic genes are labeled within the shared set. (C) GSEA of genes in BT-549 cells grown in 3D vs. 2D culture (left) and BT-549/tet-MUC1shRNA 3D cells treated with DOX vs. vehicle control (right) using the HALLMARK_GLYCOLYSIS gene signature. NES: Normalized Enrichment Score. (D) GSEA lollipop plots of multiple glycolysis-related gene sets for (i) BT-549 cells grown in 3D vs. 2D culture and (ii) BT-549/tet-MUC1shRNA 3D cells treated with DOX vs. vehicle control. (E) Heatmaps of glycolytic genes in (i) BT-549 cells grown in 3D vs. 2D culture, (ii) BT-549/tet-MUC1shRNA 3D cells treated with DOX vs. vehicle, and (iii) BT-549 3D cells treated with GO-203 vs. vehicle. (F and G) MetaPhOR analysis identifying transcriptional dysregulation of metabolic pathways in 3D vs. 2D BT-549 cells (F) and 3D BT-549/tet-MUC1shRNA cells treated with DOX vs. vehicle (G).

Having established MUC1-C dependency of TNBC CSCs, we then investigated how MUC1-C promotes TNBC cell stemness by performing RNA-seq on BT-549 S6 3D cells. Comparison of global transcriptional profiles in 3D vs. 2D cells demonstrated broad changes in gene expression (3070 DEGs; false discovery rate (FDR) < 0.10, fold change (FC) > 1.5), with 1,701 downregulated (DN) and 1,369 upregulated (UP) genes (Figure 2A). Furthermore, silencing MUC1-C in 3D cells demonstrated 1,678 DEGs with 604 downregulated and 1,074 upregulated genes (Figure 2A). Among 1,369 upregulated genes in 3D and 604 downregulated genes in MUC1-C silenced 3D cells, we identified 186 shared genes (Figure 2B, left). Within the 186 inversely regulated and overlapping genes, there was notable representation of genes related to the glycolysis pathway (Figure 2B, right). Accordingly, we assessed the top affected pathways (i) enriched in 3D cells and (ii) depleted in both MUC1-C-silenced or GO-203-treated 3D cells by gene set enrichment analysis (GSEA) and gene set variation analysis (GSVA). We found that the gene sets related to glycolysis, as well as the MYC signaling pathway, are significantly enriched in 3D and conversely repressed with MUC1-C targeting under 3D conditions (Figures 2C, S2A, and S2B). We initially focused on glycolysis and further identified by GSEA of the BT-549 3D cell datasets that multiple glycolysis-related signatures are suppressed by targeting MUC1-C genetically and pharmacologically (Figure 2D). TNBC cells have been associated with upregulation of *SLC2A1*; whereas expression of *HK2* and other genes encoding effectors in the glycolytic pathway has been largely heterogeneous.^25^ Here, we identified multiple glycolytic genes upregulated in 3D vs. 2D cells and downregulated by targeting MUC1-C, which included (i) *SLC2A1* encoding the GLUT1 glucose transporter, (ii) *PFKM/L/P* that encode isoforms of 6-phosphofructokinase necessary for generating fructose 1,6-bisphosphate, (iii) *ALDOA/C,* and (iv) *ENO1/2* encoding enzymes that catalyze the penultimate step in glycolysis^43^ (Figures 2E and S2C). In addition, we found that phosphoglycerate mutase 1 (PGAM1), an important effector of glycolysis, the oxidative PPP, and the serine biosynthesis pathway,^44^ is upregulated in 3D cells by a MUC1-C-mediated mechanism (Figures 2E and S2C). Similar results were obtained for *LDHA* and *LDHB* that are also deregulated in TNBC, but not for *LDHC,* which is expressed at low levels in BT-549 cells^45^(Figure S2C). MetaPhOR analysis of metabolic networks to further identify transcriptional dysregulation confirmed that the glycolysis pathway is upregulated in 3D (Figure 2F) and downregulated in MUC1-C silenced 3D (Figure 2G) cells.

### MUC1-C drives expression of SLC2A1/GLUT1 and HK2 in TNBC CSCs

**Figure 3:**
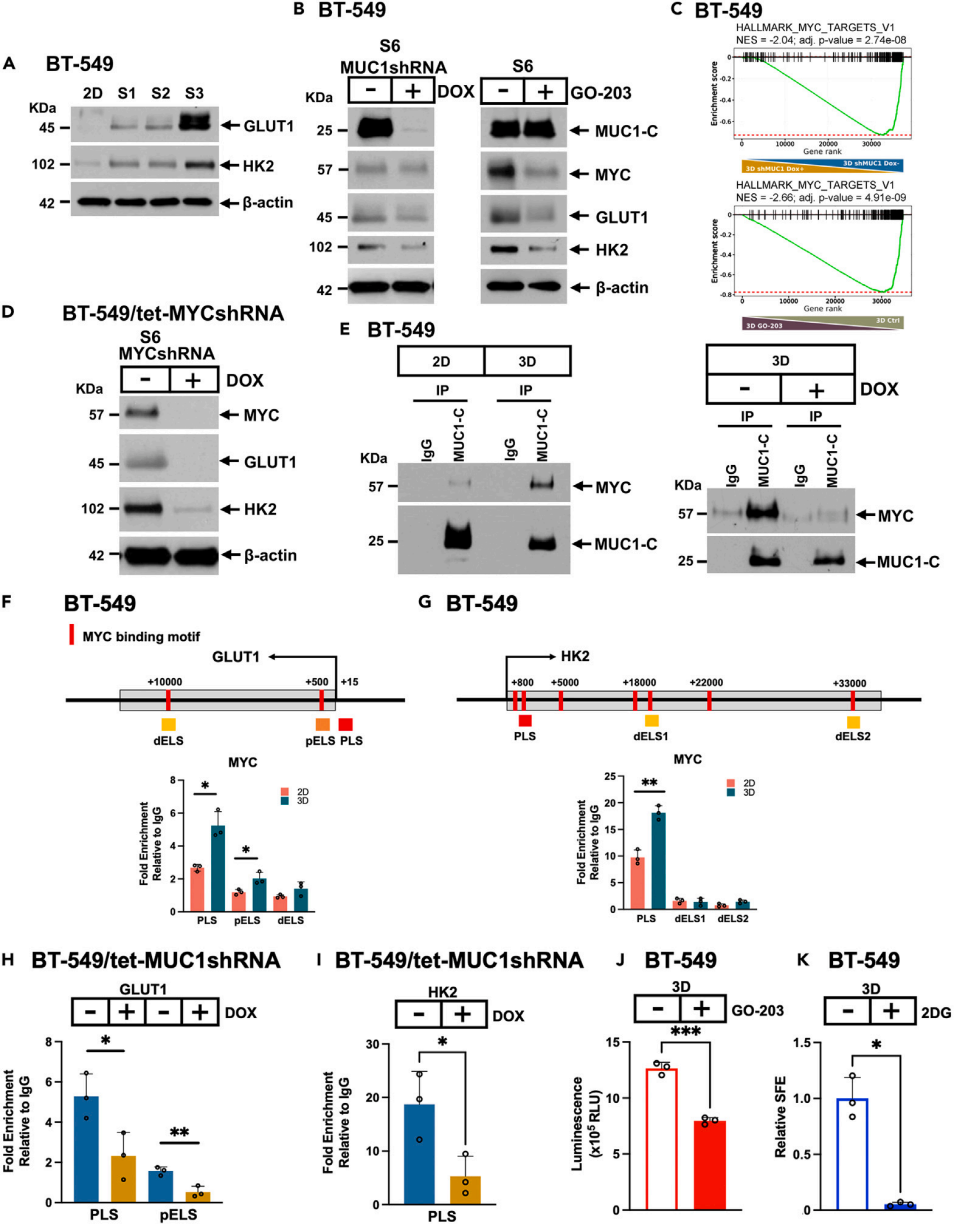
MUC1-C is necessary for induction of GLUT1 and HK2 in TNBC CSCs (A) Lysates from 2D and S1-S3 cells were immunoblotted with antibodies against the indicated proteins. (B) Lysates from BT-549/tet-MUC1shRNA S6 3D cells treated with vehicle or DOX for 10 days (left) and BT-549 cells treated with vehicle or 2.5 μM GO-203 for 2 days (right) were immunoblotted with antibodies against the indicated proteins. (C) GSEA of genes in (i) BT-549/tet-MUC1shRNA S6 cells treated with DOX vs. vehicle and (ii) BT-549 cells treated with GO-203 vs. vehicle using the HALLMARK_MYC_TARGETS_V1 gene signature. (D) Lysates from BT-549/tet-MYCshRNA S6 3D cells treated with vehicle or DOX for 10 days were immunoblotted with antibodies against the indicated proteins. (E) Nuclear lysates from BT-549 2D and 3D cells were immunoprecipitated with anti-MUC1-C or a control IgG (left). Nuclear lysates from BT-549 3D cells treated with vehicle or 2.5 μM GO-203 for 3 days were immunoprecipitated with anti-MUC1-C or a control IgG (right). The precipitates were immunoblotted with antibodies against the indicated proteins. (F) Schema of *GLUT1* with positioning of the PLS, pELS, and dELS regions. Soluble chromatin from BT-549 2D and 3D cells was precipitated with a control IgG or anti-MYC antibody. The DNA samples were amplified by qPCR with primers for the *GLUT1* PLS, pELS, and dELS regions (Table S2). The results (mean ± SD of 3 determinations) are expressed as fold enrichment relative to that obtained with the IgG control (assigned a value of 1). Asterisks represent ∗p ≤ 0.05. (G) Schema of *HK2* with positioning of the PLS, dELS1, and dELS2 regions. Soluble chromatin from BT-549 2D and 3D cells was precipitated with a control IgG or anti-MYC antibody. The DNA samples were amplified by qPCR with primers for the *HK2* PLS, dELS1, and dELS2 regions (Table S2). The results (mean ± SD of 3 determinations) are expressed as fold enrichment relative to that obtained with the IgG control (assigned a value of 1). Asterisks represent ∗∗p ≤ 0.01. (H and I) Soluble chromatin from BT-549/tet-MUC1shRNA 3D cells treated with vehicle or DOX was precipitated with a control IgG or anti-MYC antibody. The DNA samples were amplified by qPCR with primers for the *GLUT1* PLS and pELS regions (H), and *HK2* PLS region (I). The results (mean ± SD of 3 determinations) are expressed as fold enrichment relative to that obtained with the IgG control (assigned a value of 1). Asterisks represent ∗p ≤ 0.05, ∗∗p ≤ 0.01. (J) Glucose uptake was measured in BT-549 3D cells treated with vehicle or 2.5 μM GO-203 for 3 days. The results (mean ± SD of 3 determinations) are expressed as luminescence (relative light unit, RLU). Asterisks represent ∗∗∗p ≤ 0.001. (K) BT-549 3D cells were treated with vehicle or 5 μM 2DG for 7 days. The relative SFE is expressed as the mean ± SD of three determinations as compared to that obtained for vehicle-treated cells (assigned a value of 1). Asterisks represent ∗p ≤ 0.05.

Based on the importance of GLUT1 and HK2 in driving glycolysis and tumorigenicity,^25^^,^^46^ we confirmed their (i) upregulation in 3D vs. 2D cells (Figure 3A) and (ii) suppression by targeting MUC1-C with silencing and GO-203 treatment (Figures 3B and S3A). *GLUT1* and *HK2* have been recognized as MYC target genes.^47^ Along these lines, we found that targeting MUC1-C in 3D cells results in the downregulation of MYC expression (Figure 3B). Analysis of the HALLMARK_MYC_TARGETS_V1 signature further revealed significant enrichment in 3D vs. 2D cells (Figure S2A) that was abrogated by MUC1-C targeting (Figure 3C). RNA-seq data from MYC silenced 3D cells also showed significant loss of enrichment in the REACTOME_GLYCOLYSIS gene set (Figure S3B). Moreover, Landscape In Silico deletion Analysis (LISA) demonstrated enrichment of multiple MYC cistromes for many of the upregulated genes in 3D vs. 2D cells (Figure S3C). These findings supported the potential involvement of MUC1-C→MYC signaling in activating *GLUT1* and *HK2* in CSCs. In accordance with this notion and, like MUC1, silencing MYC in 3D cells suppressed expression of GLUT1 and HK2 (Figures 3D, S3D, and S3E). MUC1-C associates with MYC in total lysates obtained from cells grown in 2D culture.^48^ Here, we found that the interaction between MUC1-C and MYC in the nucleus is increased in 3D vs. 2D cells (Figure 3E, left), consistent with the observation that MUC1-C localizes to the chromatin fraction of 3D cells. The MUC1-C CQC motif binds directly to the MYC HLH-LZ domain.^48^ In support of this interaction, treatment of 3D cells with GO-203, which blocks the CQC motif, disrupted the formation of nuclear MUC1-C/MYC complexes (Figure 3E, right). The *GLUT1* promoter region includes E-boxes for potential MYC binding in a (i) promoter-like signature (PLS), (ii) proximal enhancer-like signature (pELS), and (iii) distal enhancer-like signature (dELS) (Figure 3F). ChIP studies performed on the *GLUT1* PLS, pELS, and dELS regions demonstrated increased MYC occupancy in 3D vs. 2D cells (Figure 3F). Analysis of the *HK2* promoter further identified PLS and dELS regions with E-boxes (Figure 3G). MYC occupancy was detectable on the PLS, but not the dELS, in 3D vs. 2D cells (Figure 3G). MUC1-C promotes MYC occupancy on MYC target genes in 2D cells.^48^ Here, we found that silencing MUC1-C in 3D cells decreases MYC occupancy of the (i) *GLUT1* PLS and pELS regions (Figure 3H) and (ii) *HK2* PLS (Figure 3I), in support of MUC1-C dependency in the activation of GLUT1 and HK2 expression. Notably, in contrast to *SLC2A1* and *HK2*, other MUC1-C-dependent glycolysis genes, such as *PFKL*, *ALDOA*, and *ENO2*, were upregulated by silencing MYC (Figures S3E and S3F), indicating that MUC1-C also induces genes in the glycolytic pathway by MYC-independent mechanisms. As substantiated by the involvement of MUC1-C in inducing GLUT1 and HK2, silencing MUC1-C decreased glucose uptake in CSCs (Figure 3J). Moreover, treatment with the 2-deoxy-D-glucose (2DG) HK2 inhibitor^49^ markedly decreased mammosphere formation in concordance with dependence of CSCs on MUC1-C-induced activation of the glycolytic pathway (Figure 3K).

### MUC1-C differentially regulates expression of nuclear genes encoding mitochondrial Complex I–V proteins

**Figure 4:**
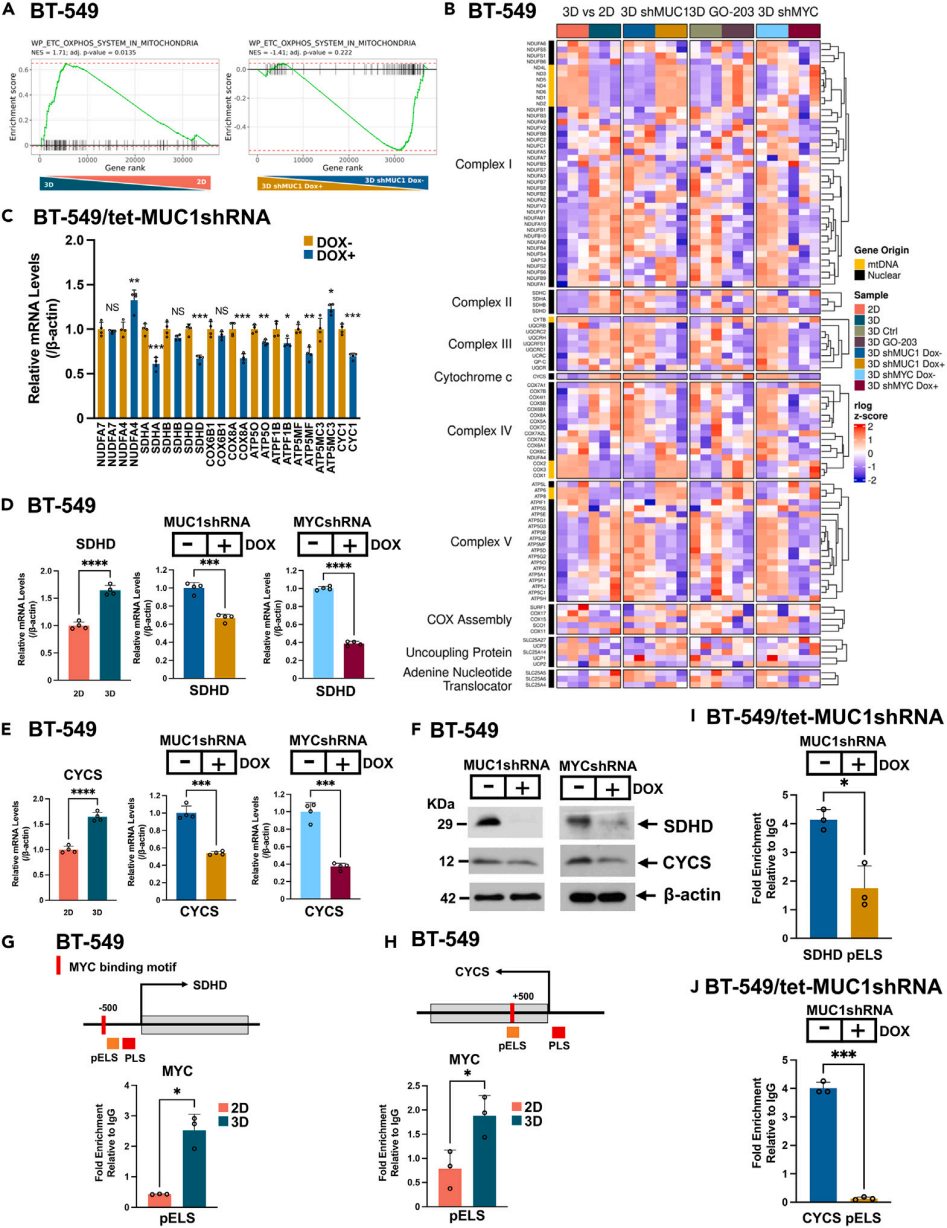
MUC1-C is necessary for expression of nuclear genes encoding components of the mitochondrial ETC (A) GSEA of genes in BT-549 cells grown in 3D vs. 2D culture (left) and BT-549/tet-MUC1shRNA 3D cells treated with DOX vs. vehicle control (right) using the WP_ETC_OXPHOS_MITOCHONDRIA gene signature. (B) WP_ETC_OXPHOS_SYSTEM IN MITOCHONDRIA signature heatmaps of ETC encoding genes in (i) BT-549 cells grown in 3D vs. 2D culture, (ii) BT-549/tet-MUC1shRNA 3D cells treated with DOX vs. vehicle, (iii) BT-549 3D cells treated with GO-203 vs. vehicle, and (iv) BT-549/tet-MYCshRNA 3D cells treated with DOX vs. vehicle. The row indicator shows gene origins, nuclear DNA (black) and mtDNA (yellow). (C) Expression of the indicated nuclear genes in BT-549/tet-MUC1shRNA 3D cells treated with DOX vs. vehicle for 7 days was determined by qRT-PCR. The results (mean ± SD of 3 determinations) are expressed as relative mRNA levels compared to that obtained for vehicle-treated cells (assigned a value of 1). Asterisks represent ∗p ≤ 0.05, ∗∗p ≤ 0.01, ∗∗∗p ≤ 0.001. (D–F) BT-549 3D vs. 2D cells and BT-549/tet-MUC1shRNA 3D cells treated with DOX vs. vehicle for 7 days were analyzed for SDHD (D) and CYCS (E) expression. The qRT-PCR results (mean ± SD of 3 determinations) are expressed as relative mRNA levels compared to that obtained for 2D cells or vehicle-treated cells (assigned a value of 1). Lysates were immunoblotted with antibodies against the indicated proteins (F). Asterisks represent ∗∗∗p ≤ 0.001, ∗∗∗∗p ≤ 0.0001. (G) Schema of *SDHD* with positioning of an E-box in the pELS region. Soluble chromatin from BT-549 2D and 3D cells was precipitated with a control IgG or anti-MYC antibody. The DNA samples were amplified by qPCR with primers for the *SDHD* pELS region (Table S2). The results (mean ± SD of 3 determinations) are expressed as fold enrichment relative to that obtained with the IgG control (assigned a value of 1). Asterisks represent ∗p ≤ 0.05. (H) Schema of *CYCS* with positioning of an E-box in the pELS region. Soluble chromatin from BT-549 2D and 3D cells was precipitated with a control IgG or anti-MYC antibody. The DNA samples were amplified by qPCR with primers for the *CYCS* PLS and pELS regions (Table S2). The results (mean ± SD of 3 determinations) are expressed as fold enrichment relative to that obtained with the IgG control (assigned a value of 1). Asterisks represent ∗p ≤ 0.05. (I and J) Soluble chromatin from BT-549/tet-MUC1shRNA 3D cells treated with vehicle or DOX for 5 days was precipitated with a control IgG or anti-MYC antibody. The DNA samples were amplified by qPCR with primers for the *SDHD* pELS (I) and *HK2* pELS regions (J). The results (mean ± SD of 3 determinations) are expressed as fold enrichment relative to that obtained with the IgG control (assigned a value of 1). Asterisks represent ∗p ≤ 0.05, ∗∗∗p ≤ 0.001.

In extending the aforementioned observation that MUC1-C plays a role in regulating glycolysis in TNBC CSCs, GSEA using the WP_ETC_OXPHOS_MITOCHONDRIA signature demonstrated enrichment in BT-549 3D vs. 2D cells (Figure 4A). We also found that silencing MUC1-C in 3D CSCs both represses and activates OXPHOS genes (Figure 4A). Furthermore, heatmaps of genes activated in BT-549 3D vs. 2D cells and regulated in (i) BT-549/tet-MUC1shRNA 3D cells treated with DOX vs. vehicle and (ii) BT-549 3D cells treated with GO-203 vs. vehicle identified specific nuclear genes encoding components of the electron transfer chain (ETC) (Figure 4B). Among these, we confirmed that silencing MUC1-C suppresses expression of (i) the succinate dehydrogenase A (SDHA) catalytic subunit of Complex II, (ii) ubiquinol-cytochrome *c* reductase complex cytochrome *c*1 (CYC1) component of Complex III, (iii) COX8A, an essential component of Complex IV cytochrome *c* oxidase and (iv) the ATP5MF component of Complex V ATP synthase^50^^,^^51^^,^^52^ (Figure 4C). In contrast, silencing MUC1-C increased expression of (i) the NDUFA4 component of Complex I that also associates with Complex IV^53^ and (ii) ATP5MC3, a subunit of Complex V ATP synthase^54^ (Figure 4C), indicating that MUC1-C differentially regulates nuclear genes encoding the ETC. MYC activates nuclear genes that play roles in mitochondrial replication and biogenesis; however, less is known about MYC function in regulating nuclear genes that encode ETC components in CSCs.^55^ We found that silencing MYC in CSCs suppresses the WP_ETC_OXPHOS_MITOCHONDRIA signature (Figure S4A). Heatmaps of BT-549/tet-MYCshRNA 3D cells treated with DOX vs. vehicle further identified nuclear genes encoding ETC components that are upregulated in 3D vs. 2D cells and suppressed by MYC silencing (Figures 4B and S4B). Among selected genes, we found that silencing MUC1-C and MYC downregulates *SDHD* (Figure 4D). A similar dependency on MUC1-C and MYC was identified for the *CYCS* gene that encodes cytochrome *c*, an essential carrier of electrons from Complex III to Complex IV (Figure 4E). By extension, MUC1-C and MYC were necessary for expression of the SDHD and cytochrome *c* proteins (Figure 4F). The *SDHD* promoter region includes an E-box for potential MYC binding in a pELS (Figure 4G). ChIP studies performed on the *SDHD* pELS region demonstrated increases in MYC occupancy in 3D vs. 2D cells (Figure 4G). Analysis of the *CYCS* promoter also identified a pELS with an E-box that was occupied by MYC at increased levels in 3D vs. 2D cells (Figure 4H). In addition, we found that silencing MUC1-C in 3D cells decreases MYC occupancy of the *SDHD* pELS (Figure 4I) and *CYCS* pELS (Figure 4J) regions. These results indicated that MUC1-C regulates nuclear genes encoding proteins necessary for function of mitochondrial Complexes I–V and that these MUC1-C-driven genes are regulated by MYC-independent and -dependent pathways.

### MUC1-C suppresses mtDNA gene expression

**Figure 5:**
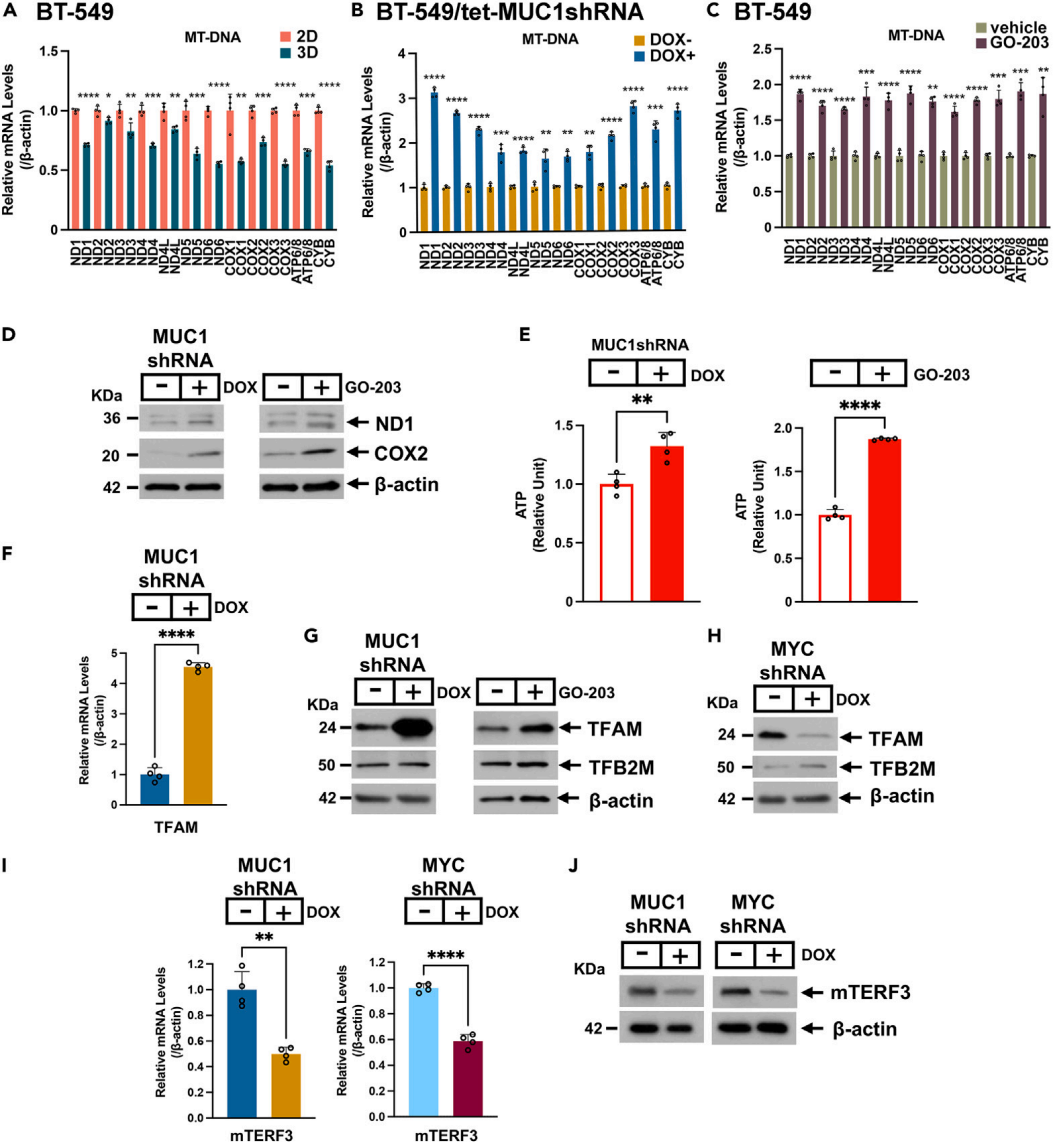
MUC1-C represses mtDNA gene expression (A) Expression of the indicated mtDNA genes in 3D vs. 2D BT-549 cells was determined by qRT-PCR. The results (mean ± SD of 3 determinations) are expressed as relative mRNA levels compared to that obtained for 2D cells (assigned a value of 1). Asterisks represent ∗p ≤ 0.05, ∗∗p ≤ 0.01, ∗∗∗p ≤ 0.001, ∗∗∗∗p ≤ 0.0001. (B and C) Expression of the indicated mtDNA genes in (B) BT-549/tet-MUC1shRNA 3D cells treated with DOX vs. vehicle for 10 days, and (C) BT-549 3D cells treated with 5 μM GO-203 vs. vehicle for 36 h was determined by qRT-PCR. The results (mean ± SD of 3 determinations) are expressed as relative mRNA levels compared to that obtained for vehicle-treated cells (assigned a value of 1). Asterisks represent ∗p ≤ 0.05, ∗∗p ≤ 0.01, ∗∗∗p ≤ 0.001, ∗∗∗∗p ≤ 0.0001. (D) Lysates from BT-549/tet-MUC1shRNA 3D cells treated with DOX vs. vehicle for 7 days (left) and BT-549 3D cells treated with 2.5 μM GO-203 vs. vehicle for 2 days (right) were immunoblotted with antibodies against the indicated proteins. (E) BT-549/tet-MUC1shRNA 3D cells treated with DOX vs. vehicle for 4 days (left) and BT-549 3D cells treated with 5 μM GO-203 vs. vehicle for 2 days (right) were analyzed for ATP levels. The results (mean ± SD of 3 determinations) are expressed as relative ATP levels compared to that obtained for vehicle-treated cells (assigned a value of 1). Asterisks represent ∗∗p ≤ 0.01, ∗∗∗∗p ≤ 0.0001. (F) BT-549/tet-MUC1shRNA 3D cells treated with DOX vs. vehicle for 5 days were analyzed for TFAM expression by qRT-PCR. The results (mean ± SD of 3 determinations) are expressed as relative mRNA levels compared to that obtained for vehicle-treated cells (assigned a value of 1). Asterisks represent ∗∗∗∗p ≤ 0.0001. (G) Lysates from BT-549/tet-MUC1shRNA 3D cells treated with DOX vs. vehicle for 7 days (left) and BT-549 3D cells treated with 2.5 μM GO-203 vs. vehicle for 2 days (right) were immunoblotted with antibodies against the indicated proteins. (H) Lysates from BT-549/tet-MYCshRNA 3D cells treated with vehicle or DOX were immunoblotted with antibodies against the indicated proteins. (I) BT-549/tet-MUC1shRNA (left) and BT-549/tet-MYCshRNA (right) 3D cells treated with vehicle or DOX were analyzed for mTERF3 expression by qRT-PCR. The results (mean ± SD of 3 determinations) are expressed as relative mRNA levels compared to that obtained for vehicle-treated cells (assigned a value of 1). Asterisks represent ∗∗p ≤ 0.01, ∗∗∗∗p ≤ 0.0001. (J) Lysates from BT-549/tet-MUC1shRNA and BT-549/tet-MYCshRNA 3D cells treated with vehicle or DOX for 7 days were immunoblotted with antibodies against the indicated proteins.

Complex I interacts with Complexes III and IV to form the “respirasome”.^51^ Complex I consists of 45 subunits, which include the essential ND1-6 components of the ETC that are encoded by mtDNA genes.^50^^,^^56^ mtDNA includes (i) the light strand promoter (LSP), which generates transcripts encoding ND6 and 8 tRNAs, (ii) the heavy strand promoter 1 (HSP1) that controls transcription of 2 tRNAs and 2 ribosomal RNAs, and (iii) HSP2, which regulates expression of the additional 12 protein-encoding transcripts (ND1-ND5, COX1/2/3, ATP6/8, and CYB) and 12 tRNAs.^57^ Analysis of the WP_ETC_OXPHOS_MITOCHONDRIA gene signature, which includes nuclear- and mtDNA-encoding genes, indicated that expression of *ND1-6*, *ND4L*, *COX1/2/3*, *ATP6/8*, and *CYB* is downregulated in 3D vs. 2D cells (Figure 4B). By extension, we confirmed uniform suppression of these 13 mtDNA genes in 3D vs. 2D cells by qRT-PCR (Figure 5A), which supported repression of the mtDNA heavy and light strands. These observed effects in 3D vs. 2D cells were unrelated to decreases in mtDNA copy number, as evidenced by the mtDNA/B2M gDNA ratio (Figure S5A). MitoTracker Green staining of 3D vs. 2D cells further demonstrated similar levels of mitochondria (Figure S5B), in accordance with other mechanisms responsible for the suppression of mtDNA gene transcription. The WP_ETC_OXPHOS_SYSTEM IN MITOCHONDRIA gene heatmap also revealed that MUC1-C is involved in mtDNA gene regulation (Figure 4B). Accordingly, we confirmed that targeting MUC1-C with silencing (Figure 5B) and GO-203 treatment (Figure 5C) induces ND1-6, ND4L, COX1/2/3, ATP6/8, and CYB expression. Targeting MUC1-C also increased (i) expression of the ND1 and COX2 proteins (Figure 5D) and (ii) ATP levels (Figure 5E), indicating that the MUC1-C-induced suppression of mtDNA gene expression is coupled with downregulation of ETC function. Mitochondrial transcription factor A (TFAM) is a nuclear gene-encoded protein that binds mtDNA and is essential for mtDNA packaging and transcription.^57^ TFAM regulates mtDNA transcription in complexes with the TF2BM transcription factor.^56^ In association with the induction of mtDNA gene transcription, we found that targeting MUC1-C induces TFAM, but not TFB2M, expression (Figures 5F, 5G, and S5C), indicating that TFAM is downregulated by a MUC1-C-mediated mechanism. Silencing MYC in 3D cells was also associated with induction of mtDNA genes (Figures S5D and S5E) and increases in ATP production (Figure S5F); however, in contrast to MUC1-C, silencing MYC clearly decreased TFAM transcripts and protein (Figures 5H and S5G). In addition, silencing MYC decreased TFB2M transcripts and had variable effects on expression of the TFB2M protein (Figures 5H and S5G). Surprisingly, we also found that silencing MUC1-C, as well as MYC, downregulates the mitochondrial transcription termination factor 3 (mTERF3) (Figures 5I and 5J), which represses the mtDNA light and heavy strands.^58^ These results supported a model in which mtDNA gene transcription is systematically repressed by (i) MUC1-C-mediated suppression of TFAM and (ii) MUC1-C/MYC-induced mTERF3 expression.

### MUC1-C suppresses mitochondrial ROS production in CSCs

**Figure 6:**
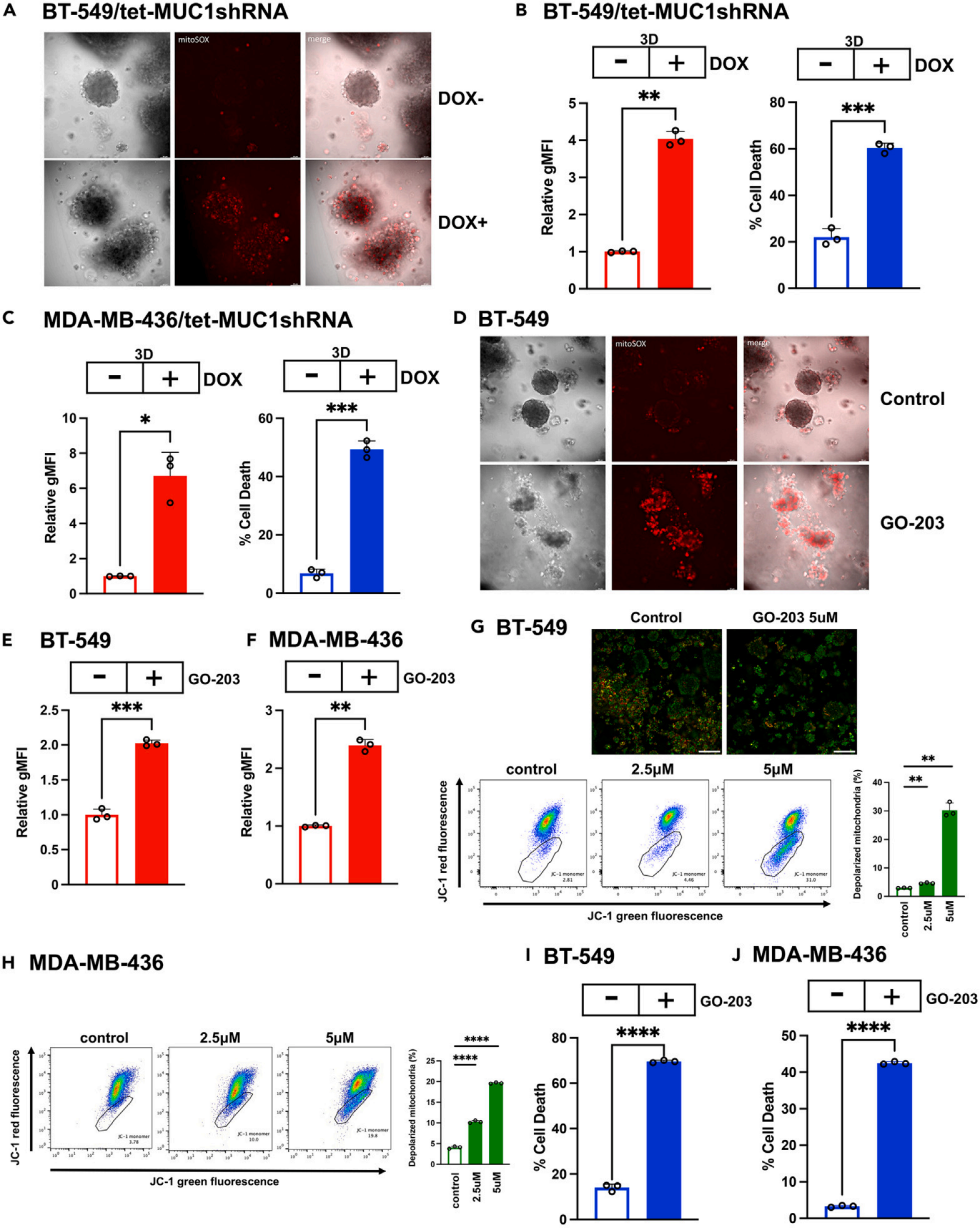
MUC1-C suppresses superoxide production in CSCs (A) BT-549/tet-MUC1shRNA mammospheres treated with vehicle or DOX for 10 days were stained with MitoSOX Red. Shown are representative fluorescence microscopy images. (B and C) BT-549/tet-MUC1shRNA (B) and MDA-MB-436/tet-MUC1shRNA (C) mammospheres were treated with vehicle or DOX for 10 days. MitoSOX Red flow cytometry data (mean ± SD of 3 determinations) are expressed as relative geometric mean fluorescence intensity (gMFI) compared to that obtained for vehicle-treated cells (assigned a value of 1)(left). Trypan blue staining results (mean ± SD of 3 determinations are expressed as the % cell death (right). (C) MitoSOX Red flow cytometry data in MDA-MB-436/tet-MUC1shRNA mammospheres treated with vehicle or DOX for 10 days. The results (mean ± SD of 3 determinations) are expressed as fold-change of gMFI for the mammosphere cells (left). MDA-MB-436/tet-MUC1shRNA mammospheres treated with vehicle or DOX for 10 days were monitored for cell death by trypan blue staining. The results are expressed as the % cell death (mean ± SD of 3 determinations)(right). Asterisks represent ∗p ≤ 0.05, ∗∗p ≤ 0.01, ∗∗∗p ≤ 0.001. (D) BT-549 mammospheres treated with vehicle or 5 μM GO-203 for 3 days were stained with MitoSOX Red. Shown are representative fluorescence microscopy images. (E and F) BT-549 (E) and MDA-MB-436 (F) mammospheres were treated with vehicle or 5 μM GO-203 for 3 days. MitoSOX Red flow cytometry data (mean ± SD of 3 determinations) are expressed as relative geometric mean fluorescence intensity (gMFI) compared to that obtained for vehicle-treated cells (assigned a value of 1). The results (mean ± SD of 3 determinations) are expressed as fold-change of gMFI for the mammophere cells. Asterisks represent ∗∗p ≤ 0.01, ∗∗∗p ≤ 0.001. (G) BT-549 mammospheres treated with vehicle or the indicated GO-203 concentrations for 3 days were stained with JC-1. Fluorescence images are shown for the control and GO-203-treated cells (upper panels). The JC-1 stained mammosphere cells were analyzed by flow cytometry for assessment of JC-1 red vs. green emission as a measure of the MMP (lower panels). The results (mean ± SD of 3 determinations) are expressed as the % depolarized mitochondria (JC-1 monomers). Asterisks represent ∗∗p ≤ 0.01. (H) MDA-MB-436 mammospheres treated with vehicle or the indicated GO-203 concentrations for 3 days were stained with JC-1. The JC-1 stained mammosphere cells were analyzed by flow cytometry for assessment of JC-1 red vs. green emission. The results (mean ± SD of 3 determinations) are expressed as the % depolarized mitochondria (JC-1 monomers). Asterisks represent ∗∗∗∗p ≤ 0.0001. (I and J) BT-549 (I) and MDA-MB-436 (J) mammospheres treated with vehicle or 5 μM GO-203 for 3 days were monitored for cell death by trypan blue staining. The results (mean ± SD of 3 determinations) are expressed as the % cell death. Asterisks represent ∗∗∗∗p ≤ 0.0001.

Superoxides are largely produced by the ETC in association with ATP production.^59^ The demonstration that MUC1-C suppresses mtDNA gene expression and ATP production indicated that MUC1-C plays a role in regulating CSC mitochondrial redox balance. In support of such involvement, mitochondrial superoxide levels as assessed by MitoSOX staining were markedly increased by silencing MUC1-C in BT-549 mammosphere cells (Figure 6A). Flow cytometry of individual BT-549 3D cells confirmed significant increases in superoxide production and loss of viability (Figure 6B). Silencing MUC1-C in MDA-MB-436 mammosphere cells was also associated with induction of superoxide levels and cell death (Figure 6C). MUC1-C-driven decreases in ROS could potentially be associated with increases in expression of the mitochondrial superoxide dismutase 2 (SOD2) and peroxiredoxin 3 (PRDX3) ROS scavengers. However, silencing MUC1-C had little if any effect on expression of these anti-oxidant enzymes (Figure S6). Targeting MUC1-C with GO-203 treatment of BT-549 mammospheres further demonstrated increases in superoxides by MitoSOX staining (Figure 6D) that were confirmed in individual CSCs by flow cytometry (Figure 6E). Similar results were obtained from MDA-MB-436 mammospheres treated with GO-203 and stained with MitoSOX (Figure 6F). Excessive ROS production is associated with disruption of the mitochondrial membrane potential (MMP) and cell death.^60^ Consistent with that association, staining with the membrane-permeant JC-1 dye demonstrated that targeting MUC1-C with GO-203 in BT-549 (Figure 6G) and MDA-MB-436 (Figure 6H) 3D cells decreases the red/green fluorescence intensity ratio as evidence of mitochondrial depolarization. Moreover, GO-203-induced loss of the MMP was associated with CSC death (Figures 6I and 6J), in support of CSC dependence on MUC1-C to maintain mitochondrial function and thereby self-renewal capacity. These findings indicate that TNBC CSCs are largely dependent on MUC1-C for suppression of mtDNA gene expression and superoxide production to maintain redox balance and survival; however, we do not exclude the possibility that they could also be attributed to dysregulation of uncoupling protein expression in mitochondria.

### MUC1-C regulates distinct subpopulations of glycolytic- and OXPHOS-enriched CSCs

**Figure 7:**
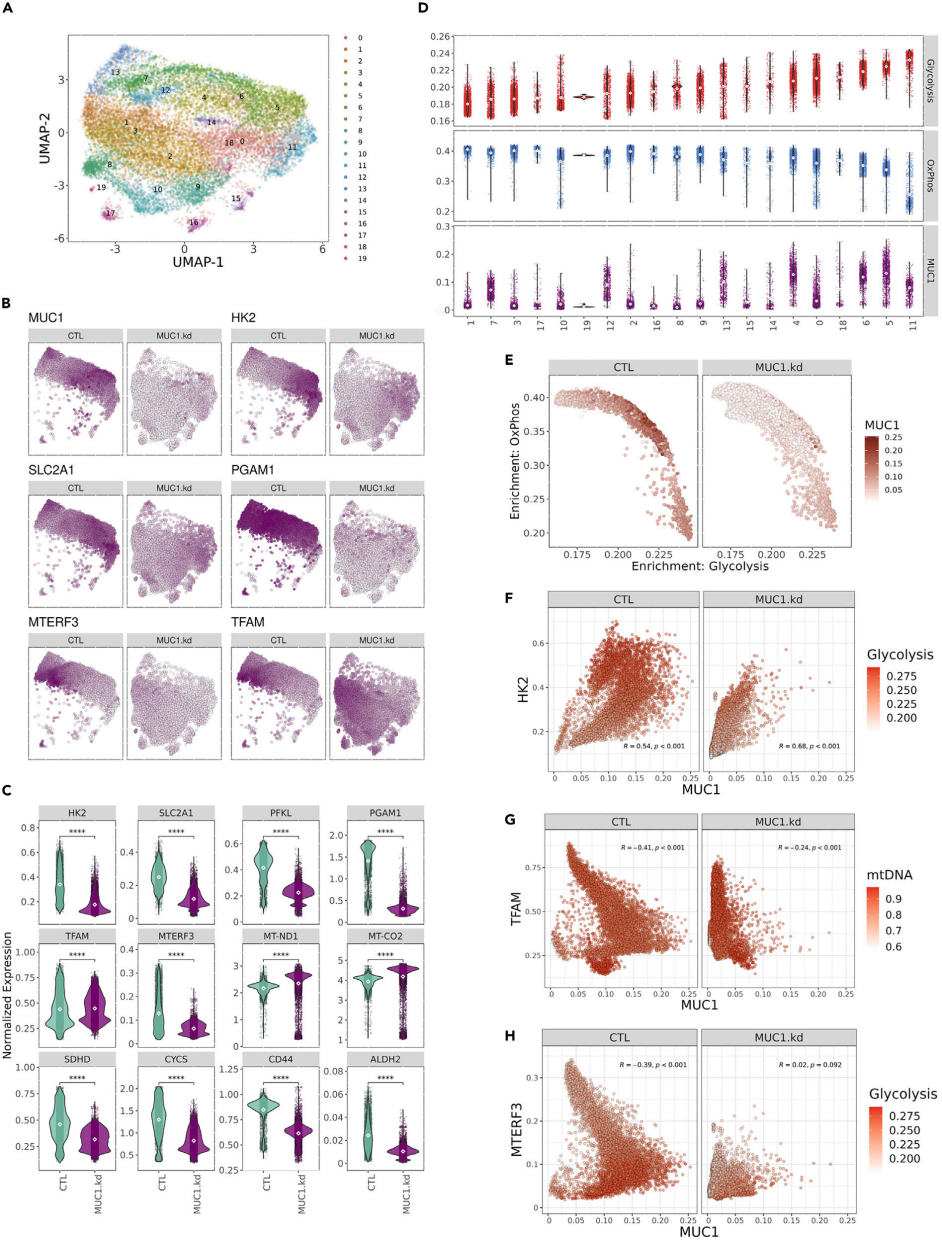
Single-cell RNA-seq identifies metabolic heterogeneity in mammosphere CSCs that associates with MUC1 expression (A) UMAP visualization of cells and clusters identified. (B) Expression feature plots of select genes associated with glycolysis (HK2, SLC2A1, and PGAM1) and transcriptional regulators of mitochondrial DNA (TFAM and MTERF3) visualized by UMAP representation reveal metabolic heterogeneity within passaged sphere cells. (C) Expression of glycolytic genes (HK2, SLC2A1, PFKL, and PGAM1), mitochondrial transcriptional regulators (TFAM and MTERF3), mitochondrial encoded components of ETC (MT-ND1 and MT-CO2), nuclear encoded components of ETC (SDHD and CYCS), and stemness genes (CD44 and ALDH2) associate with MUC1 status in passaged sphere cells. (D) Metabolic signature scores and MUC1 expression levels across clusters. (E) Comparison of single-cell HALLMARK_GLYCOLYSIS and HALLMARK_OXIDATIVE_PHOSPHORYLATION signature scores within individual cells. Color represents MUC1 expression within individual cells. (F) Correlation of HK2 and MUC1 expression across CTL or MUC1 knockdown passaged sphere cells with color intensity representing HALLMARK_GLYCOLYSIS signature scores. (G) Comparison of TFAM and MUC1 expression in CTL or MUC1 knockdown passaged sphere cells. Color represents overall signature of ETC genes encoded by the mitochondrial genome. (H) Correlation of MTERF3 expression and MUC1 expression with color intensity indicating HALLMARK_GLYCOLYSIS enrichment score.

To determine if MUC1-C dictates glycolytic and OXPHOS pathways within the same or different CSCs, we performed scRNA-seq on isolated BT-549 mammospheres. The scRNA-seq data were filtered to remove dying cells and predicted cell doublets (Figure S7A). Consistent with bulk RNA-seq findings, knockdown of MUC1-C in BT-549 mammosphere cells drives substantial transcriptomic remodeling as indicated by nearly discrete clustering of MUC1-C silenced versus control single cells (Figure S7B). Graph-based nearest neighbor clustering identified 20 unique clusters with distinct transcriptomic signatures across both samples (Figures 7A and S7C). Furthermore, differential gene expression comparing MUC1-C silenced and control conditions displayed significant concordance between bulk- and scRNA-seq analyses (Figure S7D). Expression of MUC1-C was heterogeneous across control cells compared with consistent loss in MUC1-C knockdown cells (Figure 7B). As observed in the bulk RNA-seq studies, silencing MUC1-C downregulated key glycolysis genes (*HK2*, *SLC2A1/GLUT1*, and *PFKL*) (Figures 7B and 7C). Downregulation of MUC1-C also resulted in notable, uniform suppression of the integral glycolysis PGAM1 enzyme (Figures 7B and 7C). Moreover, silencing MUC1-C increased TFAM and decreased mTERF3 expression (Figures 7B and 7C). Notably, in MUC1-C-low cells, the combination of TFAM-high/mTERF3-low expression coincided with upregulation of mtDNA encoded genes, including *ND1* and *COX2* (Figure 7C). Normalized expression confirmed that these effects of silencing MUC1-C on critical components of glycolysis and the ETC are highly significant and associated with decreases in expression of the CD44 and ALDH2 stemness markers (Figure 7C). The differential single-cell gene expression profiles provided sufficient granularity to identify distinct, and mutually exclusive, functional clusters by GSEA (Figures 7A and S7E). Clusters 5 and 6 were specifically enriched for HALLMARK glycolysis, mTORC1 signaling, and hypoxia signatures; whereas, clusters 1, 3, 7, 12, and 19 represent cells with high OXPHOS and MYC regulated HALLMARK signatures (Figures 7D and S7E). Although cluster 11 is highly enriched for a glycolytic signature, further analysis suggested that these cells may be dying as evidenced by high expression of heat shock protein, HSPA5, and long non-coding RNAs, NEAT1, and MALAT1 (Figure S7C). Consistent with the bulk RNA-seq data, loss of MUC1-C expression was associated with significant downregulation of the HALLMARK_GLYCOLYSIS signature (Figure S7F). Moreover, glycolysis and OXPHOS signatures were inversely correlated and this tendency was conserved with alterations of MUC1 expression (Figures 7D and 7E). Along these lines, further analysis revealed a significant positive association between MUC1 and HK2 expression that drives enrichment of the HALLMARK_GLYCOLYSIS signature in MUC1-high and HK2-high cells (Figure 7F). We also found that the signature of mtDNA encoding ETC genes is significantly elevated upon MUC1 knockdown in association with upregulation of TFAM and downregulation of mTERF3 expression (Figures 7G and 7H). These findings indicate that CSCs are metabolically heterogeneous and MUC1-C is a key regulator of metabolic reprogramming between the glycolysis and OXPHOS phenotypes.

## Discussion

Aerobic glycolysis and mitochondrial metabolism have been extensively studied in cancer cells growing as monolayers in 2D culture and as tumors^27^^,^^61^^,^^62^; whereas, less is known about the intersection of glycolytic and mitochondrial pathways in 3D cells. In the present work, serially passaged TNBC mammospheres were isolated as an approach for performing such studies on enriched CSCs. In this way, we found that TNBC CSC 3D vs. 2D cells are dependent on MUC1-C for (i) induction of genes encoding EMT-TFs, BMI1, SOX2, and MYC, (ii) self-renewal capacity, and (iii) tumorigenicity. MUC1-C is activated in barrier tissues by loss of homeostasis.^2^ As a result, MUC1-C induces inflammatory, remodeling, and repair responses associated with wound healing that, if prolonged as in settings of chronic inflammation, are established in promoting carcinogenesis.^2^ The wound healing response involves activation of glycolysis to maintain redox balance.^63^ We found that MUC1-C activates the glycolytic pathway in CSCs, which may relate to irreversible establishment of this pathway in the progression of chronic inflammation to cancer.^2^ Specifically, targeting MUC1-C genetically and pharmacologically in CSCs downregulated expression of *SLC2A1/GLUT1*, *HK2*, and other glycolytic genes. MUC1-C drives the CSC state in part by interacting with the MYC HLH-LZ domain that regulates MYC-mediated gene transcription.^48^ As a consequence, MUC1-C promotes binding of MYC to target genes involved in epigenetic reprogramming and chromatin remodeling.^2^^,^^10^^,^^48^^,^^64^ The present studies demonstrate that MUC1-C is necessary for MYC occupancy on the *SLC2A1/GLUT1* and *HK2* promoter regions and thereby induction of GLUT1 and HK2 expression. Direct binding of MUC1-C to MYC is dependent on the redox-regulated CQC motif in the MUC1-C cytoplasmic domain,^2^^,^^48^ indicating that MUC1-C and MYC could function in concert in the response of CSCs to changes in redox balance. Notably, however, MUC1-C-dependent, MYC-independent signaling was identified for induction of other genes, such as *PFKL*, *ALDOA*, and *ENO2*, that are essential for driving glycolysis and are upregulated in cancer.^43^ Additionally, scRNA-seq revealed that loss of MUC1-C results in marked suppression of PGAM1, which is essential for glycolytic flux and anabolic pathways of nucleotide and amino acid synthesis necessary for cancer cell biogenesis.^65^

Reports of CSC metabolic properties have been conflicting.^66^ CSCs have been associated with dependence on glycolysis and decreases in ROS levels.^67^^,^^68^^,^^69^ By contrast, CSCs have also been shown to display higher rates of oxygen consumption and ROS production compared to non-CSCs.^70^^,^^71^ Increases in mtROS, largely in the form of superoxides, result from leakage of electrons at Complexes I, III, and IV.^72^ The present results demonstrate that, analogous to the glycolytic pathway, MUC1-C regulates nuclear genes encoding effectors of Complexes I–V that are essential for OXPHOS.^50^^,^^51^^,^^52^ These findings supported a role for MUC1-C in controlling electron transfer and thereby potentially increasing mtROS levels. Intriguingly, however, we found that MUC1-C suppresses the expression of mtDNA genes encoding components of Complexes I–V. This imbalanced MUC1-C function in regulating nuclear genes encoding ETC components and repressing mtDNA genes could lead to complex misassembly, which has been linked to longevity and cancer progression.^72^ MUC1-C-dependent repression of mtDNA genes involved the light and heavy strands, consistent with a mechanism involving the mtDNA promoter region. Indeed, we found that MUC1-C suppresses expression of TFAM, a member of the HMGB family of DNA binding factors that controls mtDNA transcription initiation.^57^ Adding to the potential complexity in the regulation of effectors that control mtDNA transcription, our results further demonstrate that MUC1-C is necessary for expression of the mTERF3 terminating factor, which could cooperate with the downregulation of TFAM in repressing mtDNA gene transcription. Subsequent studies will now be needed to determine if these findings in TNBC CSCs extend to other types of cancers.

Activation of the glycolytic pathway in cancer cells has been associated with the capacity to attenuate the accumulation of ROS and thereby alleviate oxidative and replicative stress that confer resistance to treatment.^73^ In other studies of cancer cells, abrogation of the glycolytic pathway has indicated that OXPHOS is sufficient for sustaining redox and energetic requirements.^27^^,^^74^^,^^75^ Despite these apparent discrepancies, one notion is that aerobic glycolysis and OXPHOS are both of importance for cancer progression.^26^^,^^51^^,^^76^ Along this line of thinking, we found that the glycolytic and OXPHOS pathways are differentially regulated in 3D vs. 2D cells, which could hold important implications for interpreting studies of metabolic reprogramming in cancer cell models. In addition, the present findings provide support for a model in which MUC1-C regulates glycolysis and OXPHOS in distinct clusters of CSCs. In this regard, we found that CSCs are dependent on MUC1-C-induced glycolysis as evidenced by the demonstration that 2DG treatment inhibits self-renewal. We also found that CSCs are dependent on MUC1-C-driven repression of the ETC, as targeting MUC1-C with silencing and GO-203 treatment suppresses self-renewal by inducing superoxide production, loss of the MMP, and death. In summary, our findings in TNBC cells indicate that MUC1-C drives CSC self-renewal by integrating the activation of glycolysis in certain subpopulations with suppression of OXPHOS in others. Expression of MUC1 in TNBCs associates with depletion of immune effectors in the tumor microenvironment (TME).^77^^,^^78^ Aerobic glycolysis has been linked to dysregulation of the TME.^79^ Therefore, additional studies will be needed to determine if the effects of MUC1-C on glycolysis and OXPHOS contribute to immunosuppression or other alterations in the TME. Additional studies will also be needed to further assess the role of MUC1-C in coupling the CSC state with metabolic reprogramming, which may also contribute to understanding involvement of the Warburg effect in cancer progression.^61^^,^^80^

### Limitations of the study

These studies were performed on enriched populations of TNBC CSCs as defined by serial passage of mammospheres with increasing capacity for self-renewal and tumorigenicity. One limitation of this work is that the enriched CSCs may not be representative of purified CSC populations. To address this limitation in part, we performed scRNA-seq on serially passaged mammosphere cells and confirmed our results from bulk RNA-seq studies of enriched mammospheres. Another limitation is that MUC1-C-dependent upregulation of aerobic glycolysis and suppression of OXPHOS in these enriched TNBC CSC populations may not extend to CSCs selected by different methods and from other tumor types. MUC1-C has been shown to drive the CSC state across pan-cancers. Subsequent studies are needed to determine if this dependency on MUC1-C in other settings is associated with integration of aerobic glycolysis and OXPHOS to maintain CSC redox balance and thereby self-renewal capacity.

## STAR★Methods

### Key resources table

**Table:** 

REAGENT or RESOURCE	SOURCE	IDENTIFIER
**Antibodies**		

Anti-MUC1-C	Thermo Fisher Scientific	Cat # MA5-11202; RRID:AB_11000874
Anti-ZEB1	Cell Signaling Technology	Cat #3396; RRID:AB_1904164
Anti-TWIST1	Abcam	Cat #ab50887; RRID:AB_883294
Anti-BMI1	Cell Signaling Technology	Cat #6964; RRID:AB_10828713
Anti-MYC	Abcam	Cat #ab32072; RRID:AB_731658
Anti-SOX2	Cell Signaling Technology	Cat #3579; RRID:AB_2195767
Anti-β-actin	Sigma-Aldrich	Cat #A5441; RRID:AB_476744
Anti-GLUT1	Abcam	Cat #115730; RRID:AB_10903230
anti-HK2	Cell Signaling Technology	Cat #2867; RRID:AB_2232946
anti-SDHD	NOVUS Biologicals	Cat #NBP2-83506
anti-cytochrome c	Proteintech	Cat #10993-1-AP; RRID:AB_2090467
anti-ND1	Proteintech	Cat #19703-1-AP; RRID:AB_10637853
anti-COX2	Proteintech	Cat #55070-1-AP; RRID: AB_10859832
anti-TFAM	Cell Signaling Technology	Cat #8076S; RRID:AB_1094911
anti-TFB2M	Proteintech	Cat #24411-1-AP; RRID:AB_2879530
anti-mTERF3	Abcam	Cat #230232
anti-SOD2	Cell Signaling Technology	Cat #13141; RRID: AB_2636921
anti-PRDX3	Proteintech	Cat #10664-1-AP; RRID: AB_2284207

**Bacterial and virus strains**		

Lentiviral particles for MUC1 shRNA	This paper	N/A
Lentiviral particles for MYC shRNA	This paper	N/A
Lentiviral particles for MUC1 shRNA#2	This paper	N/A
Lentiviral particles for control shRNA	This paper	N/A

**Chemicals, peptides, and recombinant proteins**		

Lipofectamine 3000 Reagent	Invitrogen	Cat #L3000008
Trizol Reagent	Invitrogen	Cat #15596018
Puromycin	InvivoGen	Cat #ant-pr-1
Geneticin	Invitrogen	Cat #10131035
GO-203	This paper	N/A
2-DG	Selleck	Cat #S4701
Doxycycline	Millipore Sigma	Cat #24390-14-5
TrypLE Express	Thermo Scientific	Cat #12604013
TRIzol	Invitrogen	Cat #15596-018
Halt™ Protease Inhibitor Cocktail (100X)	Thermo Fisher Scientific	Cat #78430
Dynabeads Protein G	Thermo Fisher Scientific	Cat #10003D
Heparin Solution	Stemcell Technologies	Cat #ST-07980

**Critical commercial assays**		

Power SYBR Green PCR Master Mix	Applied Biosystems	Cat #4367659
High Capacity cDNA Reverse Transcription Kit	Applied Biosystems	Cat #4368814
DNeasy Blood & Tissue Kit	QIAGEN	Cat #69504
MammoCult Human Medium Kit	Stemcell Technologies	Cat #ST-05620
AlamarBlue Cell Viability Reagent	Thermo Scientific	Cat #DAL1100
PureLink HiPure Plasmid DNA Purification Kit	Invitrogen	Cat #K210007
TruSeq Stranded mRNA Kit	Illumina	Cat #20020594
Glucose Uptake-Glo Assay	Promega	Cat #J1341
Luminescent ATP Detection Assay Kit	Abcam	Cat #ab113849
MitoTracker Green	Cell Signaling Technology	Cat #9074
MitoSOX Red	Thermo Fisher Scientific	Cat #M36008
MitoProbe JC-1 Assay Kit	Thermo Fisher Scientific	Cat #M34152
Zombie Aqua™	BioLegend	Cat #423101
MycoAlert Mycoplasma Detection Kit	Lonza	Cat #LT07-118

**Deposited data**		

RNA-seq	NCBI GEO	GSE222377
scRNA-seq	NCBI GEO	GSE230308

**Experimental models: Cell lines**		

BT-549	ATCC	CVCL_1092
MDA-MB-436	ATCC	CVCL_0623
HEK293T	ATCC	CVCL_0063
BT-549/tet-CshRNA	This paper	N/A
BT-549/tet-MUC1shRNA	This paper	N/A
BT-549/CshRNA	This paper	N/A
BT-549/MUC1shRNA	This paper	N/A
MDA-MB-436/tet-CshRNA	This paper	N/A
MDA-MB-436/tet-MUC1shRNA	This paper	N/A
BT-549/tet-MYCshRNA	This paper	N/A

**Experimental models: Organisms/strains**		

NSG mice	Jackson Laboratory	N/A
Nude mice	Jackson Laboratory	N/A

**Oligonucleotides**		

MUC1shRNA	SIGMA	TRCN0000122938
MYCshRNA	SIGMA	TRCN0000039642
MUC1shRNA#2	SIGMA	TRCN0000430218
CshRNA	Millipore Sigma	N/A
Primers for real-time PCR, see Table S1	This paper	N/A
Primers for ChIP-PCR, see Table S2	This paper	N/A

**Software and algorithms**		

FlowJo v10.6.2	BD Biosciences	N/A
GraphPad Prism9	Dotmatics	N/A
R package	CRAN	N/A

### Resource availability

#### Lead contact

Further information and requests for resources and reagents should be directed to the lead contact, Prof. Donald Kufe, Dana-Farber Cancer Institute, 450 Brookline Ave., Boston, MA 02215, USA. E-mail: donald_kufe@dfci.harvard.edu.

#### Materials availability

The study did not generate new unique reagents and there are no restrictions to availability.

### Experimental model and study participant details

#### Cell and culture conditions

Human BT-549 BRCA1 wild-type TNBC (CVCL_1092, ATCC) cells were cultured in RPMI1640 medium (Thermo Fisher Scientific, Waltham, MA, USA) containing 10% fetal bovine serum (FBS; GEMINI BioProducts, West Sacramento, CA, USA), 100 μg/ml streptomycin, 100 U/ml penicillin and 10 μg/ml insulin. MDA-MB-436 BRCA1 mutant TNBC (CVCL_0623, ATCC) cells were cultured in RPMI1640 medium containing 10% FBS, 100 μg/ml streptomycin and 100 U/ml penicillin. Cells were treated with the MUC1-C inhibitor GO-203.^16^^,^^19^ Cell authentication was performed using short tandem repeat analysis every 3–4 months. The cells were monitored for mycoplasma contamination every 3–4 months using the MycoAlert Mycoplasma Detection Kit (Lonza, Rockland, MA, USA). Cells were maintained in culture for 3–4 months for performing experiments.

### Method details

#### Mammosphere formation assay

Cells growing in 2D culture were seeded at 2.5–5×10^3^/well in 6-well ultra-low attachment culture plates (Corning, Glendale, AZ, USA) using the MammoCult Human Medium Kit (Stemcell Technologies, Cambridge, MA, USA).^19^ Mammospheres with diameters >100 μm were counted in triplicate under an inverted microscope. Mammospheres with diameters >100 μm were isolated and disrupted in the presence of TrypLE Express (Thermo Scientific, Rockford, IL, USA) into single-cell suspensions, which were counted and then reseeded into mammocult medium for serial passage. Sphere forming efficiency (SFE) was determined as the percentage of seeded cells that formed mammospheres. Cell viability was assessed by AlamarBlue (Thermo Scientific) staining and fluorescence intensity (560 nm excitation/590 nm emission).

#### Gene silencing and rescue

MUC1shRNA (MISSION shRNA TRCN0000122938), MYCshRNA (MISSION shRNA TRCN0000039642) and a control scrambled shRNA (CshRNA)(Millipore Sigma) were inserted into pLKO-tet-puro (Plasmid #21915; Addgene, Cambridge, MA, USA; RRID:Addgene_32017).^19^ CshRNA, MUC1shRNA, MYCshRNA, MUC1shRNA#2 (MISSION shRNA TRCN0000430218) were produced in HEK293T cells (RRID:CVCL_0063).^19^ Flag-tagged MUC1-CD was inserted into pInducer20 (plasmid #44012, Addgene).^19^ Vector-transduced cells were selected in the presence of 1–2 μg/ml puromycin. Cells were treated with 0.1% DMSO as the vehicle control or 500 ng/ml doxycycline (DOX; Millipore Sigma) for inducible gene silencing.

#### Real-time quantitative reverse-transcription PCR (qRT-PCR)

Total RNA was isolated using TRIzol (Invitrogen, Carlsbad, CA, USA). cDNAs were synthesized using the High Capacity cDNA Reverse Transcription Kit (Applied Biosystems, Grand Island, NY, USA). Samples were ampli- fied using the Power SYBR Green PCR Master Mix (Applied Biosystems) and the CFX96 Touch Real-Time PCR Detection System (Bio-Rad Laboratories, Hercules, CA, USA). Primers used for qRT-PCR and gPCR are listed in Table S1.

#### Immunoblot analysis

Whole cell lysates were prepared in RIPA buffer containing protease inhibitor cocktail (Thermo Fisher Scientific, Waltham, MA, USA).^19^ Immunoblotting was performed with anti-MUC1-C (#MA5-11202, 1:100; Thermo Fisher Scientific), anti-ZEB1 (#3396, 1:1000 dilution; Cell Signaling Technology (CST), Danvers, MA, USA), anti-TWIST1 (ab50887, 1:1000 dilution; Abcam, Waltham, MA, USA), anti-BMI1 (#6964, 1:1000 dilution; CST), anti-MYC (#ab32072, 1:1000 dilution; Abcam), anti-SOX2 (#3579, 1:1000 dilution; CST), anti-GLUT1 (#ab115730, 1:100000 dilution; Abcam), anti-HK2 (#2867, 1:1000 dilution; CST), anti-SDHD (#NBP2-83505, 1:1000, NOVUS Biologicals, Centennial, CO, USA), anti-cytochrome c (#10993, 1:4000 dilution; Proteintech; Rosemont, IL, USA), anti-ND1 (#19703, 1:1000 dilution; Proteintech), anti-COX2 (#55070, 1:5000 dilution; Proteintech), anti-TFAM (#8076, 1:1000 dilution; CST), anti-TFB2M (#24411, 1:500 dilution; Proteintech), anti-mTERF3 (#ab230232, 1:1000 dilution; Abcam), anti-SOD2 (#13141, 1:1000 dilution; CST), anti-PRDX3 (#10664, 1:2000 dilution; Proteintech), and anti-β-actin (A5441; 1:50000 dilution; Sigma, St. Louis, MO, USA).

#### Mouse tumor model studies

Six-week-old female NSG mice (Jackson Laboratory, Bar Harbor, ME, USA) were injected subcutaneously into the flank with the indicated numbers of BT-549 2D cultured and passaged mammosphere cells in 100 μl of a 1:1 solution of medium and Matrigel (BD Biosciences). The mice were monitored for the appearance of palpable tumors and sacrificed when tumor volumes reached 1000 mm^3^. Alternatively, when the mean BT-549 passaged mammosphere-derived tumor volume reached ∼250 mm^3^, the mice were pair-matched into groups and treated intraperitoneally with PBS or GO-203 (12 μg/gm body weight) daily for 70 days. Tumor measurements and body weights were recorded twice per week. For serial dilution tumor initiation assays, the indicated numbers of MDA-MB-436 2D and passaged mammosphere cells were implanted into the left and right flanks, respectively, of six-week-old female nude mice (Jackson Laboratory). These studies were conducted in accordance with the ethical regulations required for approval by the Dana-Farber Cancer Institute Animal Care and Use Committee (IACUC) under protocol 03–029.

#### RNA-seq analysis

Total RNA from cells cultured in triplicate was used to generate RNA-seq datasets.^19^ Briefly, TruSeq stranded paired-end sequencing was perfomed on an Illumina NovaSeq 6000. Reads were aligned to the human reference genome (GRCh38) using the STAR aligner (version 2.7.9a)^81^ and transcripts were quantified by *featureCounts* from the Subread package (version 2.0.1).^82^ Normalization and differential expression analysis were completed with DESeq2 in the R computing environment.^83^ Differential expression rank order for Gene Set Enrichment Analysis (GSEA) was performed using the fgsea package in R.^84^ Gene set variation analysis (GSVA) was performed using the GSVA package.^19^ Hallmark and Wikipedia (WP) Gene Signatures were obtained from the Molecular Signatures Database (MSigDB). Transcriptional regulator and motif enrichment analyses were performed using epigenetic Landscape in Silico deletion Analysis (LISA).^85^

#### MetaPhOR analysis

Transcriptional dysregulation of metabolic pathways was assessed with the R package, MetaPhOR, using bulk RNA-seq DEG lists as input.^86^ Briefly, MetaPhOR scores the direction and magnitude of dysregulation for key metabolic pathways from Kyoto Encyclopedia of Genes and Genomes (KEGG) and Pathway Studio pathways using expression data. A bootstrapping method with random selection of 100,000 comparator scores was employed to assess statistical significance of dysregulated pathways.

#### Co-immunoprecipitation of nuclear proteins

Cells were washed with PBS and incubated in cell lysis buffer (10 mmol/L HEPES, pH 8.0, 1.5 mmol/L MgCl2, 0.5% NP40, and 10 mmol/L KCl) for 10 minutes at 4°C. The total cell lysates were centrifuged at 4,000 rpm for 5 minutes at 4°C and the pellets were incubated in nuclear lysis buffer (10 mmol/L HEPES pH 8.0, 1.6 mmol/L MgCl2, 0.5% NP40, 420 mmol/L NaCl, 0.2 mmol/L EDTA, and 25% glycerol) for 20 minutes at 4°C, and then sheared by passage through 20 to 26 gauge needles. After centrifugation at 13,000 rpm for 10 minutes, the supernatants were collected as nuclear lysates.^19^ Nuclear proteins were incubated with anti-MUC1-C (#MA5-11202; Thermo Fisher Scientific) at 4°C overnight and then precipitated with Dynabeads Protein G (10003D; ThermoFisher Scientific) for 2 hours at 4°C. Beads were washed twice with washing buffer (20 mmol/L Tris-HCl, pH 8.0, 0.2 mmol/L EDTA, 1.5 mmol/L MgCl_2_, 0.5% NP40, and 150 mmol/L NaCl) and once with 10% TE buffer (BM-304A; Boston BioProducts), and then resuspended in sample loading buffer.

#### Chromatin Immunoprecipitation (ChIP)

ChIP was performed on cells crosslinked with 1% formaldehyde for 5 min at 37°C, quenched with 2 M glycine, washed with PBS, and sonicated in a Covaris E220 sonicator to generate 300–600 bp DNA fragments.^19^ Immunoprecipitation was performed using a control IgG (#3900; CST), and anti-MYC (#ab32072; Abcam). Precipitated DNAs were detected by PCR using primers listed in Table S2. The immunoprecipitated DNA was quantified using SYBR-green and the CFX96 Touch Real-Time PCR Detection System (Bio-Rad).^19^ Data are reported as fold-enrichment relative to IgG levels.

#### Glucose uptake assays

Analysis of glucose uptake in CSCs was performed using the Glucose Uptake-Glo Assay according to the manufacturer’s instructions (Promega, Madison, WI, USA).

#### ATP assays

Measurement of intracellular ATP levels was performed using the Luminescent ATP Detection Assay Kit (#ab113849; Abcam) according to the manufacturer’s instructions.

#### Mitochondrial DNA quantification

Total DNA was extracted using a DNeasy Blood & Tissue Kit according to the manufacturer’s protocol (QIAGEN, Hilden, Germany). Relative mtDNA:nDNA ratio was calculated using the ΔΔCt method for the mitochondrial-encoded human tRNA-Leu(UUR) (Fwd: *CACCCAAGAACAGGGTTTGT*;_Rev: *TGGCCATGGGTATGTTGTTA*) and nuclear-encoded human B2M (Fwd: *TGCTGTCTCCATGTTTGATGTATCT*; Rev: *TCTCTGCTCCCCACCTCTAAGT*) genes.

#### Analysis of mitochondrial mass

MitoTracker Green stock solutions were diluted into prewarmed (37°C) PBS to a working concentration of 100 nM. Dead cells were stained with Zombie Aqua™ (BioLegend). Cells were analyzed by MACSQuant Analyzer 10 Flow Cytometer (Miltenyi Biotec, Waltham, MA). A total of 20,000 events were acquired for each sample. Data were analyzed with FlowJo v10.6.2 (BD Biosciences) software. Fluorescence was measured using the standard emission filters for green (FL-1 channel) fluorescence photomultipliers.

#### Analysis of mitochondrial superoxide levels

Cells were incubated with pre-warmed MitoSOX Red staining solution diluted in HBSS to a final concentration of 5 μM for 15 min at 37°C. MitoSOX Red stained mammospheres were mixed with Matrigel, placed on 35 mm glass-bottom dishes and analyzed using a Leica THUNDER Imager 3D Cell Culture microscope. For flow cytometry analysis, MitoSOX Red-stained mammosphere cells were washed and labeled with Zombie Aqua™ (BioLegend). Cells were analyzed by MACSQuant Analyzer 10 Flow Cytometer (Miltenyi Biotec). A total of 20,000 events were acquired for each sample. Data were analyzed with FlowJo v10.6.2 (BD Biosciences) software. Fluorescence was measured using the standard emission filters for red (FL-2 channel) fluorescence photomultipliers.

#### Analysis of mitochondrial membrane potential

Mammospheres were stained with 2 μM JC-1 (Thermo Fisher Scientific) for 30 min at 37°C. JC-1 stained mammospheres were mixed with Matrigel, placed on 35 mm glass-bottom dishes and analyzed using a Leica THUNDER Imager 3D Cell Culture microscope. For flow cytometry analysis, JC-1-stained mammosphere cells were washed and labeled with Zombie Aqua™ (BioLegend). Emission spectral overlap was corrected by compensation using carbonylcyanide m-chlorophenylhydrazone (CCCP). Cells were analyzed by MACSQuant Analyzer 10 Flow Cytometer (Miltenyi Biotec). A total of 20,000 events were acquired for each sample. Data were analyzed with FlowJo v10.6.2 (BD Biosciences) software. Fluorescence was measured using the standard emission filters for green (FL-1 channel) red (FL-2 channel) fluorescence photomultipliers.

#### Analysis of mammosphere CSC populations by scRNA-seq

Pelleted single-cell suspensions were washed in 1,000 ul of PBS plus 0.4% BSA, transferred to low-retention microcentrifuge tubes (Fisher Scientific, Hampton, NH, USA), and then centrifuged for 10 minutes at 300 g at 4°C. Pellets were resuspended in 800 ul of PBS plus 0.4% BSA, and cells were counted by eye using INCYTO C-Chip Neubauer Improved Disposable Hemacytometers (VWR International Ltd., Radnor, PA, USA). A total of > 10,000 cells per sample were loaded per channel of the Chromium Next GEM Chip K for processing on the 10x Chromium Controller (10x Genomics, Pleasanton, CA, USA) followed by cDNA generation and library construction, as per manufacturer’s instructions (Chromium Next GEM Single Cell 5ʹ Reagent Kits v2 User Guide, Rev E). Libraries were normalized and pooled for sequencing on an Illumina NextSeq 500 system using a 150 cycle Mid-Output flow cell (Illumina, Inc., San Diego, CA, USA) with run parameters 26, 10, 10, 90. Sequenced libraries were demultiplexed using *cellranger mkfastq* (Cell Ranger v6.1.1). As part of the *cellranger count* pipeline, demultiplexed libraries were aligned to the human transcriptome (excluding introns) based on the 10X Genomics pre-computed human reference, *GRCh38-2020-A*. Both raw and cell-associated gene expression feature matrices (cell barcodes x gene counts) were output for use in downstream analyses.

#### scRNA-seq raw data processing, quality control, and subsequent analyses

Raw sequence data demultiplexing, barcode processing, alignment (GRCh38) and filtering for true cells were performed using the Cell Ranger Single-Cell Software Suite (v6.0.0), yielding 23,535 cells (shCTL: 8,561 cells, shMUC1: 14,974 cells) with a mean of 22,239 reads/cell (90.15% mapping rate), median of 1,175 genes/cell, 19,831 total unique detectable genes, and 2,697 median UMI counts/cell. Subsequent filtering for high quality cells, and downstream analyses were performed using Seurat (v4)^87^ (Figure S7A). Genes expressed in less than 3 cells and cells that express less than 300 genes were excluded from further analyses. Additional filtering of cells was determined based on the overall distributions of total RNA counts (<80,000) and the proportion of mitochondrial genes (<10%) detected to eliminate potential doublets and dying cells, respectively. Additional detection of doublets was performed using Scrublet.^88^ Thresholding for doublet detection was set based on total distribution of doublet scores (doublet threshold = 0.25). Quantification of mitochondrial and ribosomal gene expression was calculated using the PercentageFeatureSet function, using gene sets compiled from the HUGO Gene Nomenclature Committee database. Ultimately, 6,806 cells were removed (28.92% of total cells) after quality control assessment, and 16,729 high quality cells (shCTL: 5,837 cells, shMUC1: 10,892 cells) were included in downstream analyses. Normalization and variance stabilization were conducted using regularized negative binomial regression (sctransform) implemented with Seurat. Principle component analysis (PCA) was performed on normalized data and optimal dimensionality of the dataset was decided by examination of the Elbow plot, as the total number of PCs where gain in cumulative variation explained was greater than 0.1% (PCs = 50). The FindNeighbors function was utilized that implements a graph based nearest neighbor clustering approach, and the FindClusters function was used to identify final cell clusters (n = 20) using a resolution of 0.08. UMAP was applied for non-linear dimensional reduction to obtain a low dimensional representation of cellular states. Differential expression between clusters or samples was determined using the MAST method^89^ via the FindMarkers function, using a minimum expression proportion of 25% and a minimum log fold change of 0.25 after correction for mitochondrial and ribosomal gene proportions. Cluster specific gene set enrichment was determined using enrichR, using the collection of HALLMARK pathway signatures.^90^ Single-cell gene set enrichment was performed by UCell,^91^ using gene sets reflecting glycolytic (HALLMARK_GLYCOLYSIS) and oxidative phosphorylation (WP_ETC_OXPHOS_MITOCHONDRIA) activity. Gene expression imputation was performed using MAGIC,^92^ with parameters knn = 15, t = 3.

### Quantification and statistical analysis

#### Statistical analysis

Each experiment was performed at least three times. Unpaired two-tailed Student’s t-tests were used to assess differences between the mean ± SD of two groups. p-values were considered significant at p < 0.05. Adjusted p values derived from the Benjamini-Hochberg method identified differentially expressed genes at a false discovery rate (FDR) < 0.1 for bulk RNA sequencing data. GraphPad Prism9 was used for all statistical analyses. Asterisks represent ∗p ≤ 0.05, ∗∗p ≤ 0.01, ∗∗∗p ≤ 0.001, ∗∗∗∗p ≤ 0.0001 with CI = 95%.

## Data Availability

• Total RNA-seq and Single-cell RNA-seq data have been deposited at GEO and are publicly available as of the date of publication. Accession number is listed in the key resources table. • This paper does not report original code. • Any additional information required to reanalyze the data reported in this paper is available from the lead contact upon request. • Total RNA-seq and scRNA-seq data reported here are available from the NCBI Gene Expression Omnibus (GEO) under accessions GSE222377 and GSE230308, respectively.
