# Supplementary material for: MUC1-C integrates aerobic glycolysis with suppression of oxidative phosphorylation in triple-negative breast cancer stem cells
Source: iScience. 2023 Oct 11;26(11):108168. doi: 10.1016/j.isci.2023.108168 (PMC10616323; doi:10.1016/j.isci.2023.108168)
Supplement: Document S1. Figures S1–S7 and Tables S1 and S2 [file mmc1.pdf]

iScience, Volume *26*

Supplemental information

MUC1-C integrates aerobic glycolysis with suppression of oxidative phosphorylation in triple-negative breast cancer stem cells

Nami Yamashita, Henry Withers, Yoshihiro Morimoto, Atrayee Bhattacharya, Naoki Haratake, Tatsuaki Daimon, Atsushi Fushimi, Ayako Nakashoji, Aaron R. Thorner, Emily Isenhart, Spencer Rosario, Mark D. Long, and Donald Kufe

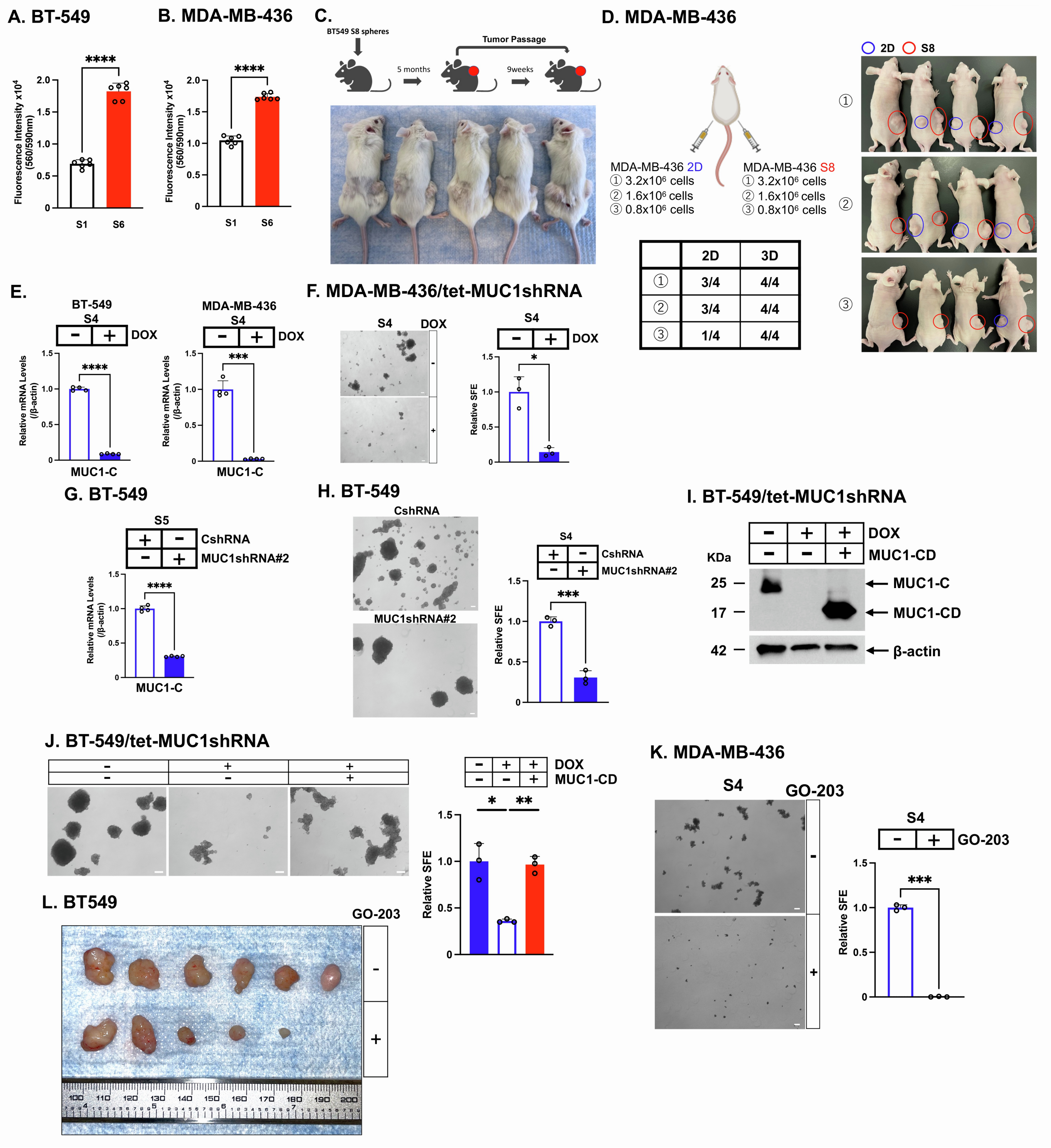

Supplemental Figure S1. Targeting MUC1-C genetically and pharmacologically inhibits CSC self-renewal capacity and tumorigenicity, Related to Figure 1. A and B. Proliferation of BT-549 (A) and MDA-MB-436(B) S1 and S6 sphere cells was determined on day 7 by Alamar Blue staining. The results (mean±SD of six determinations) are expressed as relative fluorescence intensity (560 nm excitation/590 nm emission) as compared to that obtained for S1 cells (assigned a value of 1). C. BT-549 S8 sphere cells (10x106) implanted into the flanks of NSG mice formed tumors after 5 months. Fragments (3

mm^3) of those tumors implanted into NSG mice formed tumors after 9 weeks. D. The indicated numbers of MDA-MB-436 2D and S8 mammosphere cells were implanted in to the left and right flanks, respectively, of nude mice. Listed are the numbers of tumors formed/4 mice (left). Shown are the tumors at 3 months after implantation (right). E. BT-549/tet-MUC1shRNA (left) and MDA-MB-436/tet-MUC1shRNA (right) 3D sphere cells treated with vehicle or DOX for 7 days were analyzed for MUC1-C mRNA levels by qRT-PCR. The results (mean±SD of 3 determinations) are expressed as relative mRNA levels compared to that obtained for vehicle-treated cells (assigned a value of 1). F. MDA-MB-436/tet-MUC1shRNA 3D sphere cells were treated with vehicle or DOX for 7 days. Shown are photomicrographs of representative mammospheres. Scale bar: 100 µm. (left). The relative SFE is expressed as the mean±SD of three determinations as compared to that obtained for vehicle-treated cells (assigned a value of 1)(right). G. BT-549/tet-MUC1shRNA 3D sphere cells treated with vehicle or DOX for 7 days were analyzed for MUC1-C mRNA levels by qRT-PCR. The results (mean±SD of 3 determinations) are expressed as relative mRNA levels compared to that obtained for CshRNA cells (assigned a value of1). H. BT-549/vector and BT-549/MUC1shRNA#2 cells were analyzed for sphere formation. Shown are photomicrographs of representative mammospheres (left). The relative SFE is expressed as the mean±SD of three determinations as compared to that obtained for CshRNA cells(assigned a value of 1) (right). I and J. BT-549/tet-MUC1shRNA cells expressing tet-MUC1-CD were treated with vehicle or DOX for 7 days. Lysates were immunoblotted with antibodies against the indicated proteins (I). Shown are photomicrographs of representative mammospheres (J, left). The relative SFE is expressed as the mean±SD of three determinations as compared to that obtained for vehicle-treated cells (assigned a value of 1) (right) (J, right). K. MDA-MB-436 3Dsphere cells were treated with vehicle or 5 µM GO-203 for 7 days. Shown are photomicrographs of representative mammospheres (left). The relative SFE is expressed as the mean±SD of three determinations as compared to that obtained for vehicle-treated cells (assigned a value of 1) (right). L. NSG mice with established BT-549 3D cell tumors were treated intraperitoneally each day with PBS or GO-203 (12 µg/gm bodyweight) each day for 70 days. Shown are the tumors from the control and GO-203-treated groups harvested on day 70.

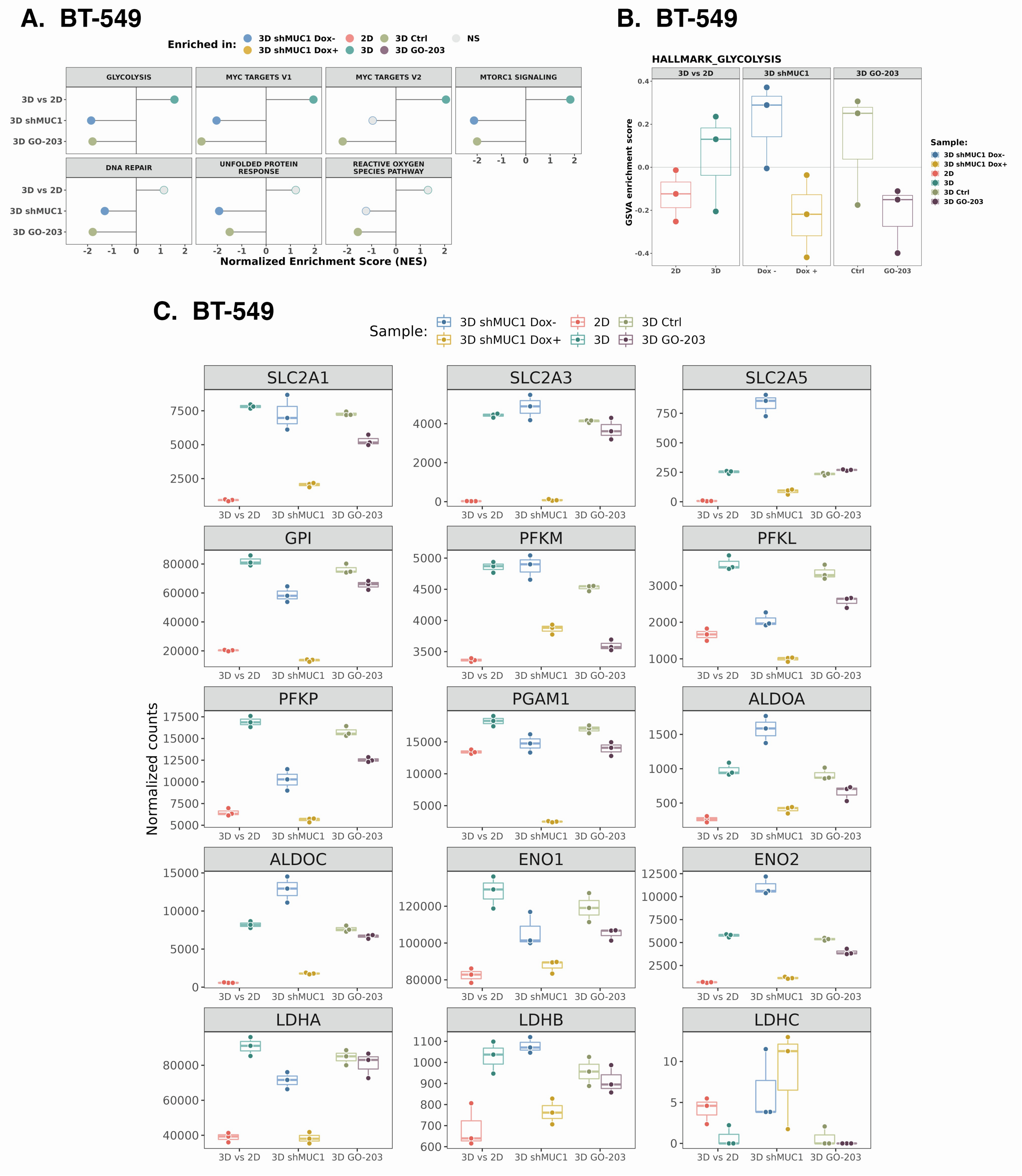

Supplemental Figure S2. MUC1-C drives glycolysis and OXPHOS genes in CSCs, Related to Figure 2. A. GSEA lollipop plots of the indicated gene sets for (i) BT-549 cells grown in 3D vs 2D culture, (ii) BT-549/tet-MUC1shRNA 3Dcells treated with DOX vs vehicle, and (iii) BT-549 3D cells treated with GO-203 vs vehicle with NS (grey) denoting FDR > 0.1. NES: Normalized Enrichment Score. B. GSVA for sample-wise enrichment of the HALLMARK_GLYCOLYSIS gene signature. C. Normalized expression counts of glycolytic genes in (i) BT-549 cells grown in 3D

vs 2D culture, (ii)BT-549/tet-MUC1shRNA 3D cells treated with DOX vs vehicle, and (iii)BT-549 3D cells treated with GO-203 vs vehicle (biological triplicates per condition).

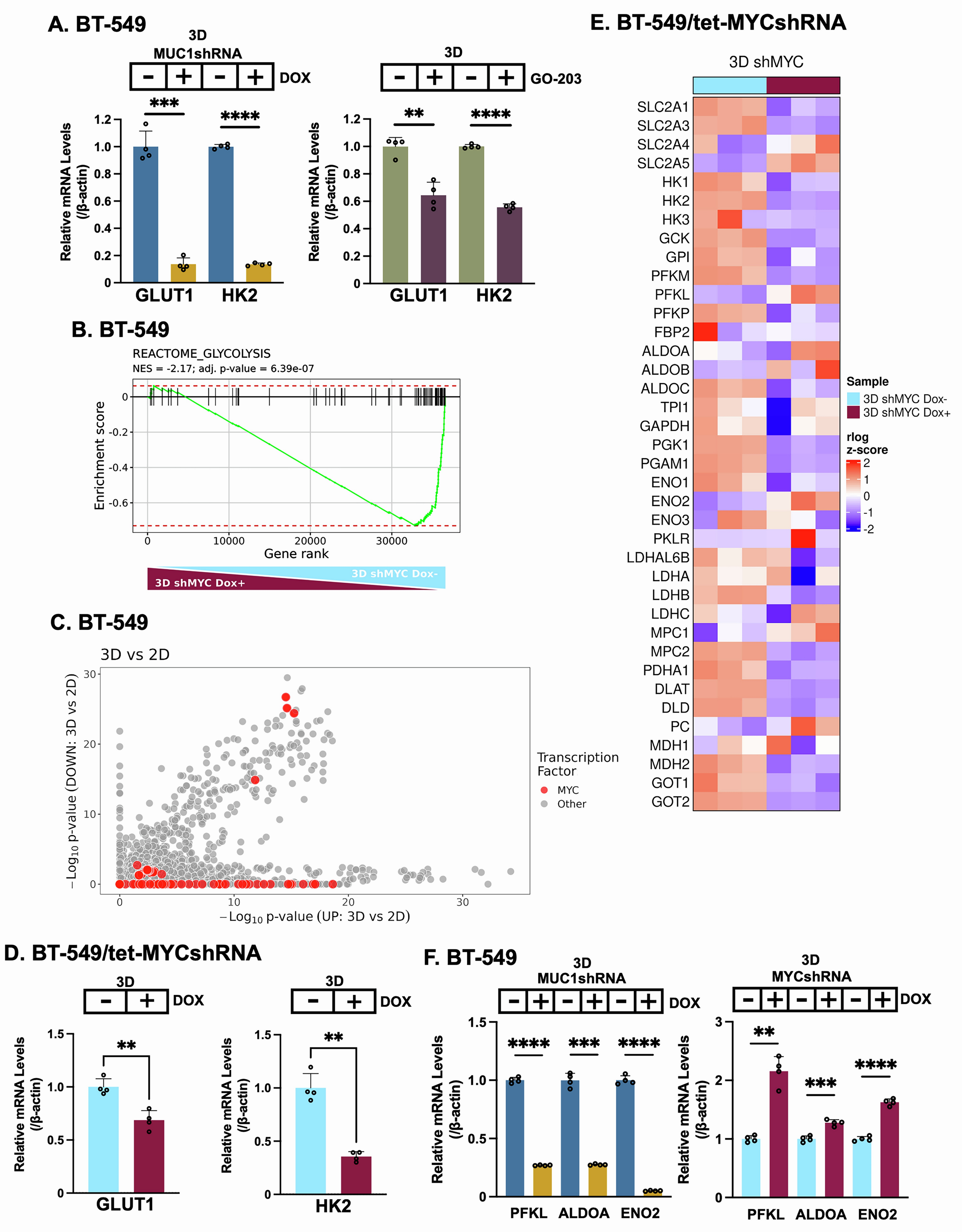

Supplemental Figure S3. MUC1-C signaling induces glycolytic genes in 3D CSCs, Related to Figure 3. A. BT-549/tet-MUC1shRNA 3D cells

treated with vehicle or DOX for 7 days (left) and BT-549 cells treated with vehicle or 2.5 μM GO-203 for 36 hours (right) were analyzed for GLUT1 and HK2 mRNA levels by qRT-PCR. The results (mean±SD of 3 determinations) are expressed as relative mRNA levels compared to that obtained for vehicle-treated cells (assigned a value of 1). B. GSEA of genes in BT-549/tet-MYCshRNA 3D cells treated with DOX vs vehicle using the REACTOME_GLYCOLYSIS gene signature. C. Epigenetic Landscape in Silico deletion Analysis (LISA) was applied to the top 500 downregulated and upregulated DEGs in the 3D vs 2D cell expression dataset. Each datapoint represents the significance level of regulatory potential of a given transcriptional factor derived from a unique ChIP-seq dataset. MYC regulated cistromes are highlighted in red. D. BT-549/tet-MYCshRNA 3D cells treated with vehicle or DOX for 5 days were analyzed forGLUT1 and HK2 mRNA levels by qRT-PCR. The results (mean±SD of 3determinations) are expressed as relative mRNA levels compared to that obtained for vehicle-treated cells (assigned a value of 1). E.Heatmaps of row-scaled rlog transcript counts for glycolytic pathway genes in BT-549/tet-MYC shRNA 3D cells treated with DOX vs vehicle. F.BT-549/tet-MUC1shRNA 3D cells treated with vehicle or DOX for 7 days(left) and BT-549/tet-MYC shRNA cells treated with vehicle or DOX for 5 days (right) were analyzed for the indicated mRNA levels by qRT-PCR. The results (mean±SD of 3 determinations) are expressed as relative mRNA levels compared to that obtained for vehicle-treated cells(assigned a value of 1).

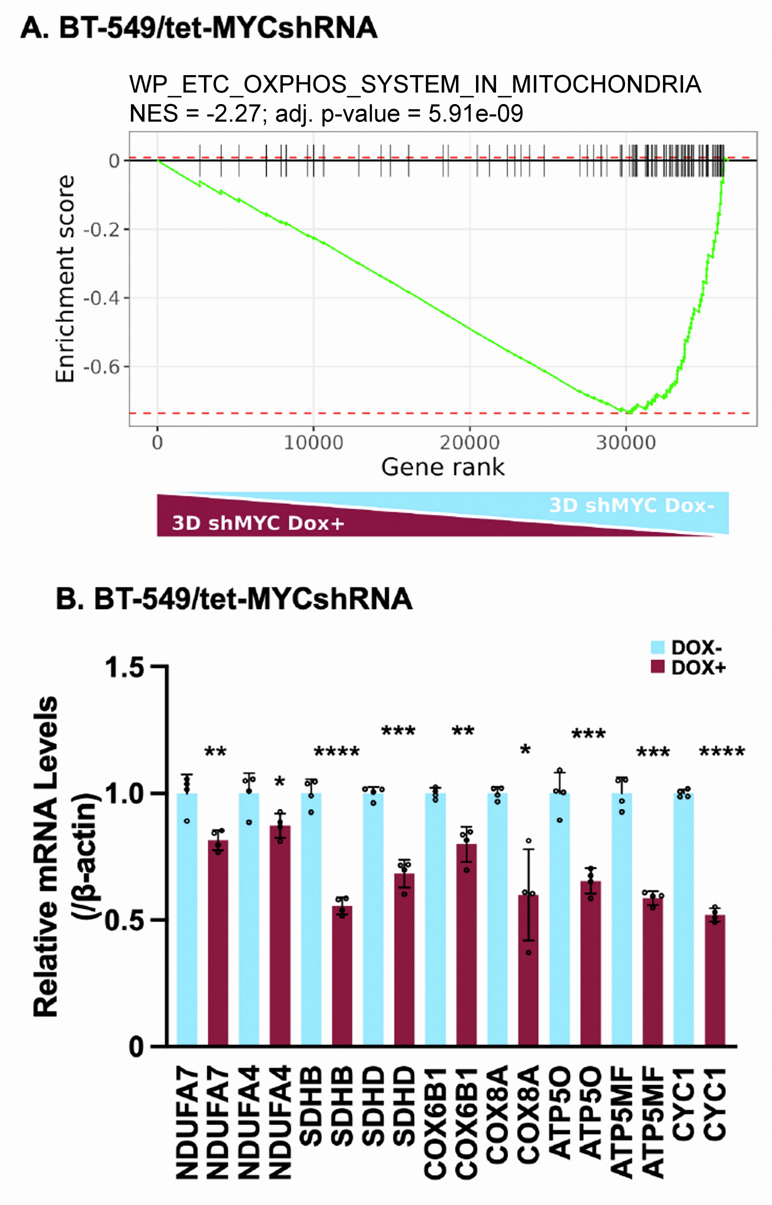

Supplemental Figure S4. Effects of silencing MYC on nuclear genes encoding components of Complexes II-V, Related to Figure 4. A. GSEA of genes in BT-549/tet-MYC shRNA 3D cells treated with DOX vs vehicle using the WP_ETC_OXPHOS_MITOCHONDRIA gene signature. B. BT-549/tet-MYC shRNA 3Dcells treated with DOX vs vehicle were analyzed for expression of the indicated nuclear genes by qRT-PCR. The results (mean±SD of 3determinations) are expressed as relative mRNA levels compared to that obtained for vehicle-treated cells (assigned a value of 1).

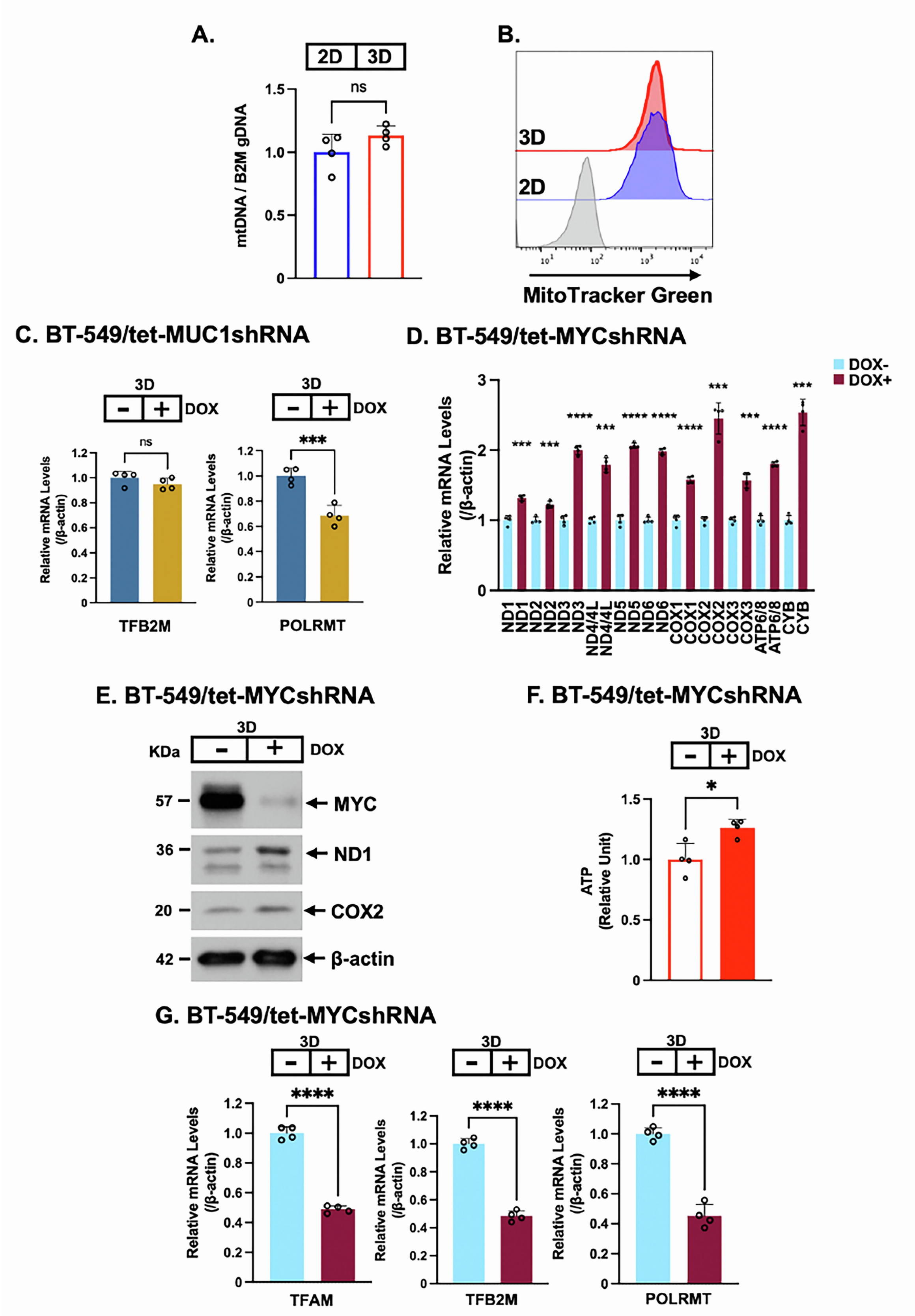

Supplemental Figure S5. Effects of MUC1-C and MYC on mtDNA gene expression, Related to Figure 5. A. Ratio of mtDNA (tRNA-Leu(UUR)) to nuclear genome DNA9(B2M) (mtDNA/B2M gDNA) in BT-549 2D vs 3D cells. B.

Fluorescence histogram of BT-549 2D vs 3D cells stained with MitoTracker Green. C. Expression of the nuclear TFB2M and POLRMT genes in BT-549/tet-MUC1shRNA 3D cells treated with vehicle or DOX was determined by qRT-PCR. The results (mean±SD of 3 determinations) are expressed as relative mRNA levels compared to that obtained for vehicle-treated cells (assigned a value of 1). D. Expression of the indicated mtDNA genes in BT-549/tet-MYCshRNA 3D cells treated with DOX vs vehicle was determined by qRT-PCR. The results (mean±SD of 3 determinations) are expressed as relative mRNA levels compared to that obtained for vehicle-treated cells (assigned a value of 1). E. Lysates from BT-549/tet-MYC shRNA 3D cells treated with vehicle or DOX were immunoblotted with antibodies against the indicated proteins. F. BT-549/tet-MYC shRNA 3D cells treated with vehicle or DOX were analyzed for ATP levels. The results (mean±SD of 3 determinations) are expressed as relative ATP levels compared to that obtained for vehicle-treated cells (assigned a value of 1). G. Expression of the nuclear TFAM, TFB2M and POLRMT genes in BT-549/tet-MYC shRNA 3D cells treated with vehicle or DOX was determined by qRT-PCR. The results (mean±SD of 3determinations) are expressed as relative mRNA levels compared to that obtained for vehicle-treated cells (assigned a value of 1).

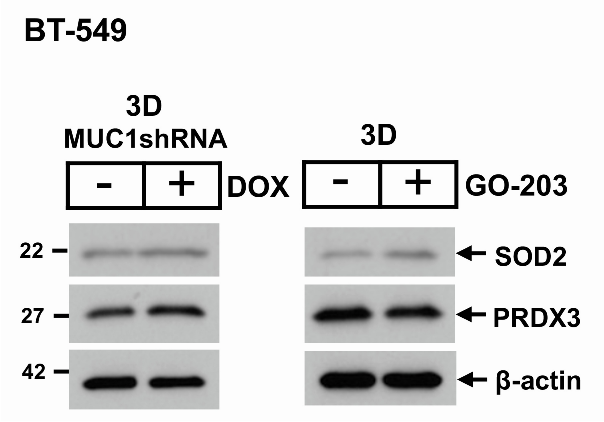

Supplemental Figure S6. Effects of MUC1-C on SOD2 and PRDX3 expression, Related to Figure 6. BT-549/tet-MUC1shRNA 3D cells treated with vehicle and DOX and BT-549 cells treated with vehicle and GO-203 were immunoblotted with antibodies against the indicated proteins.

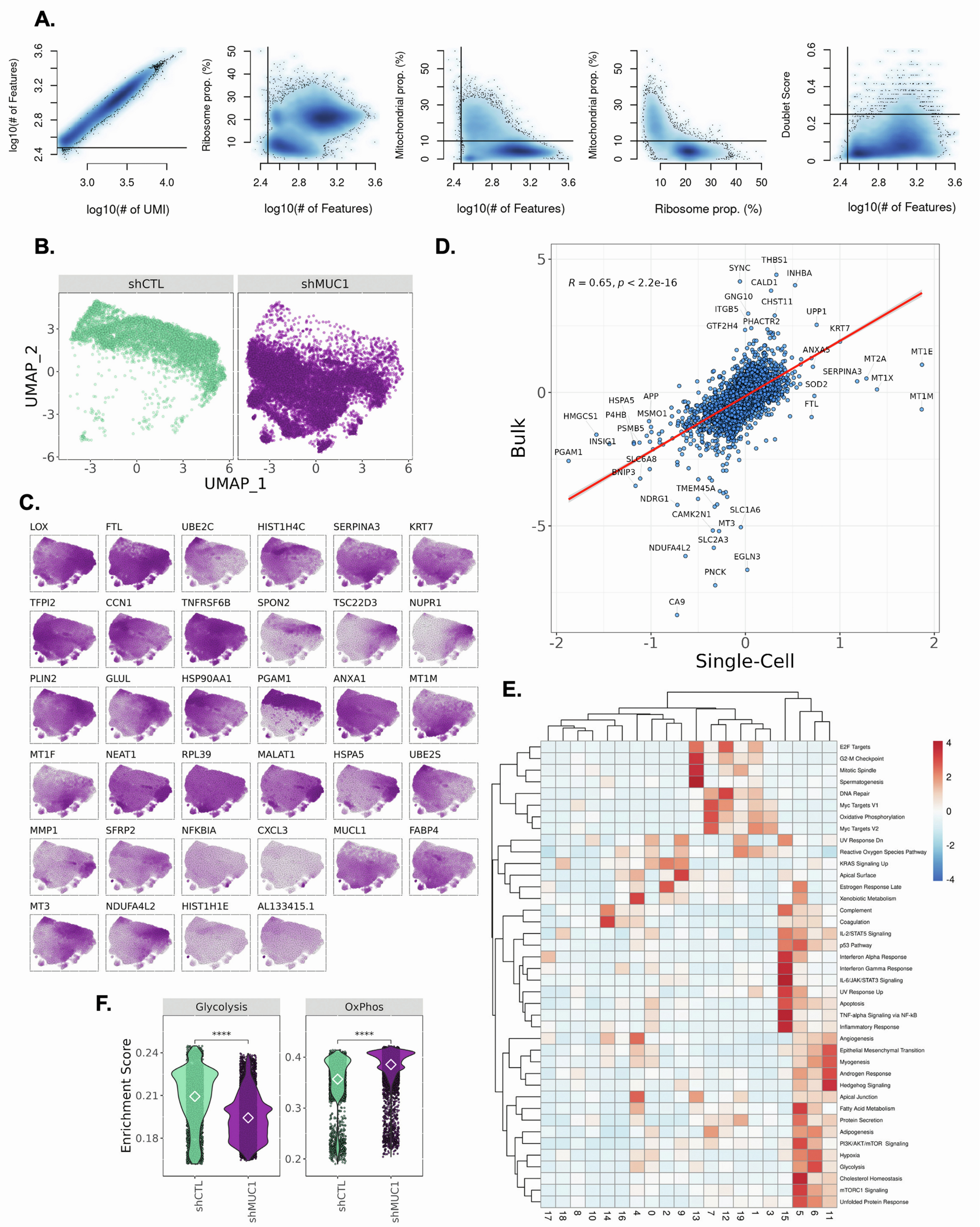

Supplemental Figure S7. Identifying transcriptional heterogeneity in CSCs using scRNA-seq, Related to Figure 7. A. Cell quality criteria

(mitochondrial expression < 10%, features > 300, Doublet Score < 0.25) used for12filtering high quality cells for downstream analysis. B. Cells separated by MUC1 status. C. Top two variable gene markers identified across clusters, visualized by UMAP representation of imputed expression. D. Correlative analysis of differential gene expression comparing MUC1-C knockdown in single-cell and bulk RNA-seq studies. E. Functional enrichment of cluster specific gene markers against all HALLMARK gene sets. F. Metabolic signature scores across all cells within CTL and MUC1-C knockdown (kd) cells.

Supplemental Table S1. Primers used for qRT-PCR, related to Figures 4, 5 and S3-5.

Primer	FWD	REV
**MUC1-C**	AGACGTCAGCGTGAGTGATG	GCCAAGGCAATGAGATAGAC
**GLUT1**	GGCCAAGAGTGTGCTAAAGAA	ACAGCGTTGATGCCAGACAG
**HK2**	TGCCACCAGACTAAACTAGACG	CCCGTGCCCACAATGAGAC
**PFKL**	GTACCTGGCGCTGGTATCTG	CCTCTCACACATGAAGTTCTCC
**ALDOA**	ATGCCCTACCAATATCCAGCA	GCTCCCAGTGGACTCATCTG
**ENO2**	CCGGGAACTCAGACCTCATC	CTCTGCACCTAGTCGCATGG
**NUDFA7**	TGCAGCTACGCTACCAGGA	GGAGGCTGAGTTCGCTTGG
**NUDFA4**	ATGCTCCGCCAGATCATCG	TGCCAGACGCAAGAGATACAG
**SDHA**	CAAACAGGAACCCGAGGTTTT	CAGCTTGGTAACACATGCTGTAT
**SDHB**	GACACCAACCTCAATAAGGTCTC	GGCTCAATGGATTTGTACTGTGC
**SDHD**	CATCTCTCCACTGGACTAGCG	TCCATCGCAGAGCAAGGATTC
**COX6B1**	CTACAAGACCGCCCCTTTTGA	GCAGAGGGACTGGTACACAC
**COX8A**	TTTGGCTTCCTGACCTTGG	GGCAACGAATGGATCTTGG
**ATP5O**	ATTGAAGGTCGCTATGCCACA	GCTTTTCACTTTAATGGAACGCT
**ATPF1B**	GGTGAATATGACCATCTCCCAG	GACAGTACAGAGGACAAAGACC
**ATP5MF**	CTTCTGGAGGTCAAACTGGG	ACCTCTTTGAAACGCTCC
**ATP5MC3**	CCCTTTGCCTGTTTGATTTCC	AAGCACTGATTCTGATGGAG
**CYC1**	AGCTATCCGTGGTCTCACC	CCGCATGAACATCTCCCCATC
**CYCS**	CTTTGGGCGGAAGACAGGTC	TTATTGGCGGCTGTGTAAGAG
**ND1**	CCACCTCTAGCCTAGCCGTTTA	GGGTCATGATGGCAGGAGTAAT
**ND2**	CTATCTCGCACCTGAAACAAGC	GGTGGAGTAGATTAGGCGTAGG
**ND3**	CCACAACTCAACGGCTACATAG	CACTCATAGGCCAGACTTAGGG
**ND4/4L**	CTAGGCTCACTAAACATTCTA	CCTAGTTTTAAGAGTACTGCG
**ND5**	TCTACCCTAGCATCACACACCG	GTTGAGGTGATGATGGAGGTGG
**ND6**	CAAACAATGTTCAACCAGTAACCACTAC	ATATACTACAGCGATGGCTATTGAGGA
**COX1**	GACGTAGACACACGAGCATATTTCA	AGGACATAGTGGAAGTGAGCTACAAC
**COX2**	AGAACCAGGCGACCTGCGAC	CCCCCGGTCGTGTAGCGGTG
**COX3**	CACTGGCCCCCAACAGGCAT	AGTATCAGGCGGCGGCTTCG
**ATP6/8**	TAGCCATACACAACACTAAAGGACGA	GGGCATTTTTAATCTTAGAGCGAAA

**CYB**	TCCTACACATCGGGCGAGGCC	GGTGATTCCTAGGGGGTTGT
**TFAM**	ATGGCGTTTCTCCGAAGCAT	TCCGCCCTATAAGCATCTTGA
**TFB2M**	CCAAGGAAGGCGTCTAAGGC	CTTTCGAGCGCAACCACTTTG
**POLRMT**	GGACTCCCCGGCAAAGAAG	CGCCACATCCACCCTGTTC
**mTERF3**	ATATCCTCTGACAATTGCT	GAATGATCCACATAGTCTCG
**β-actin**	GATGAGATTGGCATGGCTTT	CACCTTCACCGTTCCAGTTT

Supplemental Table S2. Primers used for ChIP-qPCR, related to Figures 3 and 4.

Primer	FWD	REV
**GLUT1 PLS**	TGCATACCCATCTCAAACCTG	CACCATAGACTCACCTGAACTG
**GLUT1 pELS**	TGCATACCCATCTCAAACCTG	CACCATAGACTCACCTGAACTG
**GLUT1 dELS**	GGTAGAGCTGTTTCTGATGGAG	TCTCCTAGCCCAGTTTATCTCC
**HK2 PLS**	CCCTCCAAATCAGCCTCG	TCAAGTAATCCAGGAACGCG
**HK2 dELS1**	AGAGGTAAAAGCGGAACTGG	CATTACCCTCATCTCTTGTTCCC
**HK2 dELS2**	ACCATGAGTAGAACACGCTG	TCCAACCCCAGCATGATAAC
**SDHD-pELS**	TGAGTATCTTTTCTACGGGCAC	GTAAAACAGGGAAGATTTGGGC
**SDHD-PLS**	CTCTCGACTTCCGGTTCAC	ACTTAGGCGACAATTCCCAC
**CYCS-pELS**	ATCATGGTCACGAAGTCACAG	GGGAGAGGTGGCTTTACATTG
**CYCS-PLS**	AGTATCAGTTCTTGGGCGTG	CTGACAAGATGGTAGCTAGGAG
